# The Influence of Surface Damage on Miniplates: A Study of Bacterial Attachment Across Various Strains

**DOI:** 10.12688/f1000research.159954.1

**Published:** 2025-02-04

**Authors:** Bramasto Purbo Sejati, Tetiana Haniastuti, Ahmad Kusumaatmaja, Maria Goreti Widyastuti

**Affiliations:** 1 Departemnt of Oral and maxillofacial Surgery, Universitas Gadjah Mada, Yogyakarta, Special Region of Yogyakarta, Indonesia; 2 Department of Oral Biology, Universitas Gadjah Mada, Yogyakarta, Special Region of Yogyakarta, Indonesia; 3 Department of Physics, Faculty of Mathematics and Natural Sciences, Universitas Gadjah Mada, Yogyakarta, Special Region of Yogyakarta, Indonesia

**Keywords:** surface damage, bacterial attachment, contact angle

## Abstract

**Background:**

Miniplates are frequently used in oral and maxillofacial surgery to address malocclusion issues. However, surface damage to miniplates is a significant concern that can affect surgical outcomes and patient quality of life. This study aims to evaluate the influence of miniplate surface damage on bacterial attachment, which may lead to postoperative infections.

**Methods:**

Miniplates with varying degrees of surface damage were used in this study. The damaged surfaces were subjected to special treatments to simulate postoperative conditions. Various bacterial strains, including Staphylococcus aureus, Pseudomonas aeruginosa, and Streptococcus mutans, were tested. Each type of bacteria was cultured on different miniplates for specific durations, and bacterial attachment was subsequently measured and analyzed.

**Results:**

Surface damage to miniplates significantly influenced bacterial attachment. Miniplates with more severe surface damage exhibited higher levels of bacterial attachment compared to undamaged miniplates. Furthermore, the type of bacteria impacted attachment levels, with certain strains demonstrating higher adhesion than others.

**Conclusion:**

Surface damage to miniplates increases the risk of postoperative infections due to enhanced bacterial attachment. Therefore, maintaining the integrity of miniplates during and after orthognathic surgery is crucial. Further research is necessary to develop prevention and management strategies for postoperative infections related to miniplate surface damage.

## Introduction

Miniplates have become standard in orthognathic surgery and facial reconstruction within oral and maxillofacial surgery. ^1– 3^ Miniplates are essential for the repair of face bone injuries, offering support and promoting effective bone healing. Nonetheless, the danger of infection linked to miniplates persists as a considerable consequence. Miniplates are frequently modified by bending and twisting to get a best fit and stability that conforms to the shapes of face bones. This modification enables direct contact between the miniplate and bone, hence improving stability and fostering healing. Notwithstanding the prevalent application of these adjustments, limited research has explicitly examined their influence on the risk of miniplate-associated illnesses. ^4, 5^

Debris accumulation and microbiological proliferation may arise between the miniplate and the bone when the miniplate is deformed or contorted. Moreover, modifications to the miniplate’s physical structure can impact pressure distribution and blood flow in the surgical region, thereby affecting the immune response and healing process. ^6^ Therefore, a comprehensive understanding of the correlation between miniplate adaptation and infection risk is essential for enhancing the success rates of facial surgical interventions. ^3, 7^

Complications related to osteosynthetic plates, especially in the mandible, may need their removal to reduce the risks of infection or plate failure. The pressure applied to titanium plates in the jaw during mastication heightens the risk of infection, presenting distinct problems in maxillofacial and oral contexts. ^2, 5, 8^ Surgical wounds that come into contact with oral secretions and biofilms are prone to bacterial colonization, resulting in problems like osteitis, bone necrosis, and compromised bone healing.

Biofilm-associated bacteria provide significant challenges as they provoke an immune response and demonstrate resistance to antimicrobial therapies, obstructing treatment attempts. Numerous bacterial species, such as Streptococcus, Prevotella, Staphylococcus, and Veillonella, are commonly detected in infected osteosynthesis locations. ^3, 5, 8^ Certain bacterial strains are related with minimal osteosynthetic material, whereas others, including E. faecalis, P. mirabilis, and P. aeruginosa, correlate with greater quantities of material. These strains not only heighten infection risks but also facilitate the development of multidrug resistance in patients undergoing oral and maxillofacial surgery.

This study aims to examine the influence of modifications to osteosynthetic plates on bacterial attachment. The osteosynthetic plates utilized in this study were obtained from patients due to infection or rejection and subsequently cultivated with microorganisms linked to osteosynthesis-related illnesses. The research aims to assess the clinical surface attributes of miniplates, concentrating on their hydrophobic qualities. Furthermore, the quantity of bacterial colonies will be measured, and the dispersion of bacteria on osteosynthetic plates will be delineated.

## Methods

This research focused on the collection of infected miniplates from patients at Temanggung Regional Hospital from 2020 to 2023. This study adheres to the STROBE reporting guideline. This study’s inclusion criteria mandated that patients possess simple fractures treated with titanium alloy miniplates from the Osteomed system, specifically 1.6 mm and 2.0 mm models from Acumed, USA. The miniplates were required to be non-locking or adaptation plates, with or without extended plates, in the maxillofacial region, and must have undergone adaptation through bending, with or without twisting. The miniplates must have been implanted for over two weeks and demonstrate clinical signs of infection, including exposed miniplates, pus in the surrounding area, elevated leukocyte counts, and non-union evident in radiological imaging. Miniplates that could be easily removed without substantial difficulty and without necessitating burring of the bone were also included.

The exclusion criteria removed patients under 17 years of age or over 65 years, individuals with infections associated with medically compromised conditions, patients with comminuted, infected, or multiple fractures, and those whose surgical procedures exceeded two hours in duration. Additionally, patients who did not comply with postoperative instructions, especially concerning antibiotic use, were excluded.

According to the established criteria, 12 infected miniplates were identified from a total of 651 miniplates implanted throughout the study period. A total of 12 miniplates were collected from 10 patients among 492 treated with miniplates. The study involved ten adaptation-type Osteomed miniplates: seven 1.6 mm miniplates (consisting of two four-hole L-shaped miniplates, two five-hole curved miniplates, and three four-hole straight miniplates) and three 2.0 mm miniplates of the four-hole extended type.

This study was approved by the Ethics Committee of the Faculty of Dentistry – Prof. Soedomo Dental Hospital, Universitas Gadjah Mada on July 16, 2024 with a number: 150/UN1/KEP/FKG-RSGM/EC/2024. In addition, this study adhere to the Declaration of Helsinki ( https://www.wma.net/policies-post/wma-declaration-of-helsinki-ethical-principles-for-medical-research-involving-human-subjects/).

### Macro photography of miniplates using a camera

The macro photography process utilizes a DSLR or mirrorless camera with a macro lens, exemplified by the Nikon Z50 paired with the Nikon AFS 60mm f/2.8 G Micro-Nikkor lens. Diffused natural or artificial lighting was configured using a minimum of two light sources positioned at 45-degree angles to the miniplate to achieve uniform illumination and reduce shadows. Camera settings were optimized with an aperture range of f/8 to f/11 to achieve adequate depth of field, a shutter speed between 1/125 and 1/250 seconds for precise exposure, and an ISO setting of 100 to 400 to ensure optimal clarity.

The miniplate was precisely placed within the camera’s viewfinder or LCD screen, occupying around 70% of the frame to achieve optimal composition. A minimum of 10 photographs were captured from multiple perspectives, including top-down, side, and angled views, to ensure thorough documentation. Images were examined on a computer monitor at 100% magnification, and the clearest, most detailed photographs were chosen for analysis. Surface texture, defects, and dimensions were meticulously documented for comprehensive record-keeping.

### Assessment of contact angle on miniplates

The contact angle serves as a crucial parameter for evaluating wettability and surface properties. Measurement is conducted using specialized instruments, including a contact angle goniometer. The angle formed at the liquid-solid interface tangent to the miniplate surface reflects the liquid’s spreading and adhesion characteristics. A 3 μl droplet of liquid was deposited onto the miniplate surface, and the droplet profile image was recorded using a custom device linked to a digital camera. Two formulas were utilized to determine the contact angle from the drop profile image: the linear gradient equation and the tangential line method.

### Bacterial attachment assessment

*Preparation of microorganisms and inoculum*

Strains of S. mutans (ATCC 25175), P. aeruginosa, S. aureus (ATCC 25933), and E. faecalis were obtained from the Integrated Research Laboratory at the Faculty of Dentistry, Universitas Gadjah Mada, Indonesia. A single colony of S. mutans, P. aeruginosa, S. aureus, or E. faecalis was cultured in BHI broth medium at 37°C for 24 hours. The turbidity of the bacterial suspension was adjusted to 0.5 McFarland, corresponding to 1.5 × 10 ^8^ CFU/mL. One milliliter of the bacterial suspension was introduced into 9 mL of BHI broth (1.10493.0500, Merck, Germany) medium within a Petri dish. The miniplate was immersed in the culture medium and incubated at 37°C for durations of 24 and 48 hours. Phosphate-buffered saline (PBS) was employed to rinse the adhered biofilm. The biofilm was stained with 0.1% crystal violet for 15 minutes, followed by washing with PBS, and subsequently treated with 96% ethanol to elute the bound crystal violet. The absorbance of the released crystal violet in ethanol was quantified at OD540 nm utilizing a spectrophotometer (Thermo Scientific, USA).

### Miniplate imaging utilizing Scanning Electron Microscopy (SEM)

Twelve infected miniplate samples and five unused control plates were analyzed using a Quanta 200 SEM (FEI, Oregon, USA). The plates were thoroughly dried prior to imaging due to the high vacuum conditions of the SEM. The plates were first removed from their containers, followed by the drainage of formalin and rinsing in buffer. Dehydration was conducted using a graded ethanol series, with each concentration (30%, 50%, 70%, 95%, and absolute ethanol) applied for 15 minutes, followed by three rinses in absolute ethanol. The plates underwent critical point drying utilizing a CPD 030 Critical Point Drier (Balzer, Leica, Solms, Germany) to reduce the potential for damage to fragile organic material. The plates were mounted onto aluminum SEM stubs with carbon tabs (Agar Scientific, Stansted, England) and subsequently sputter-coated with gold-palladium utilizing a Polaron E5100 SEM Coating Unit (Quorum Technology, East Grinstead, England) before imaging. The examination of plate and screw surfaces concentrated on pinpointing regions susceptible to biofilm formation, including surface protrusions, scratches, screw threads, screw hole depressions, and blood clots. Each patient sample necessitated approximately 5 hours for systematic evaluation and documentation based on the scanned photographic images.

### Statistical analysis

The biofilm formation inhibition assay was replicated independently a minimum of five times. The results are expressed as the mean ± standard deviation (SD) from one representative experiment. A Kruskal-Wallis test, followed by a post hoc Mann-Whitney test, was performed to evaluate the significance between groups. Statistical analysis utilized SPSS software, version 16.0, with a significance threshold established at p < 0.05.

## Result

### Macro photography of miniplates

**Figure 1.**
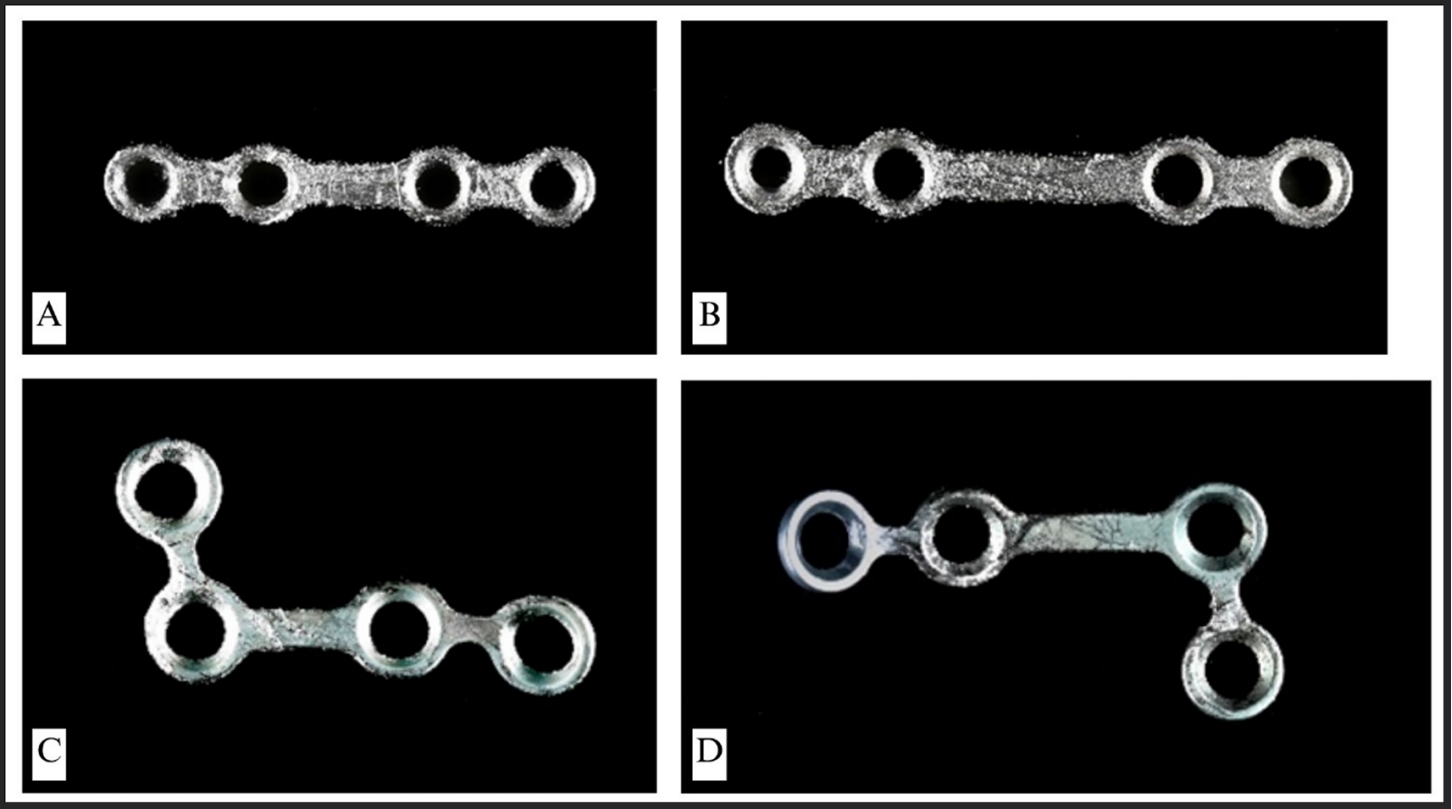
Macro photograph of the rejected miniplates. (A) and (B) Straight-type BSSO miniplates, (C) and (D) L-shaped type miniplates.

A macro image of the infected or rejected miniplates is presented ( Figure 1). A variety of plates were gathered, comprising straight-type BSSO plates and L-shaped plates. All collected plates displayed discernible surface irregularities and abnormalities. The bridge section of the straight-type BSSO plates exhibited the greatest deformation, presumably due to recurrent bending and twisting in this region. L-shaped plates had evident deformities and surface irregularities, with the most prominent distortion occurring on two sides of the plates.

### Calculation of contact angle on miniplates

**Figure 2.**
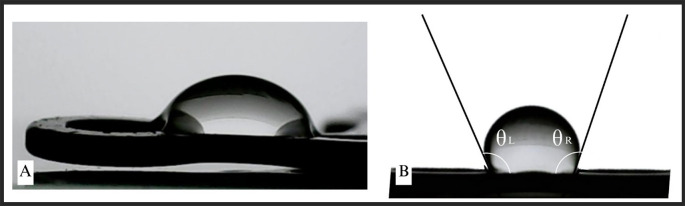
Contact angle measurement on the miniplates.

**Figure 3.**
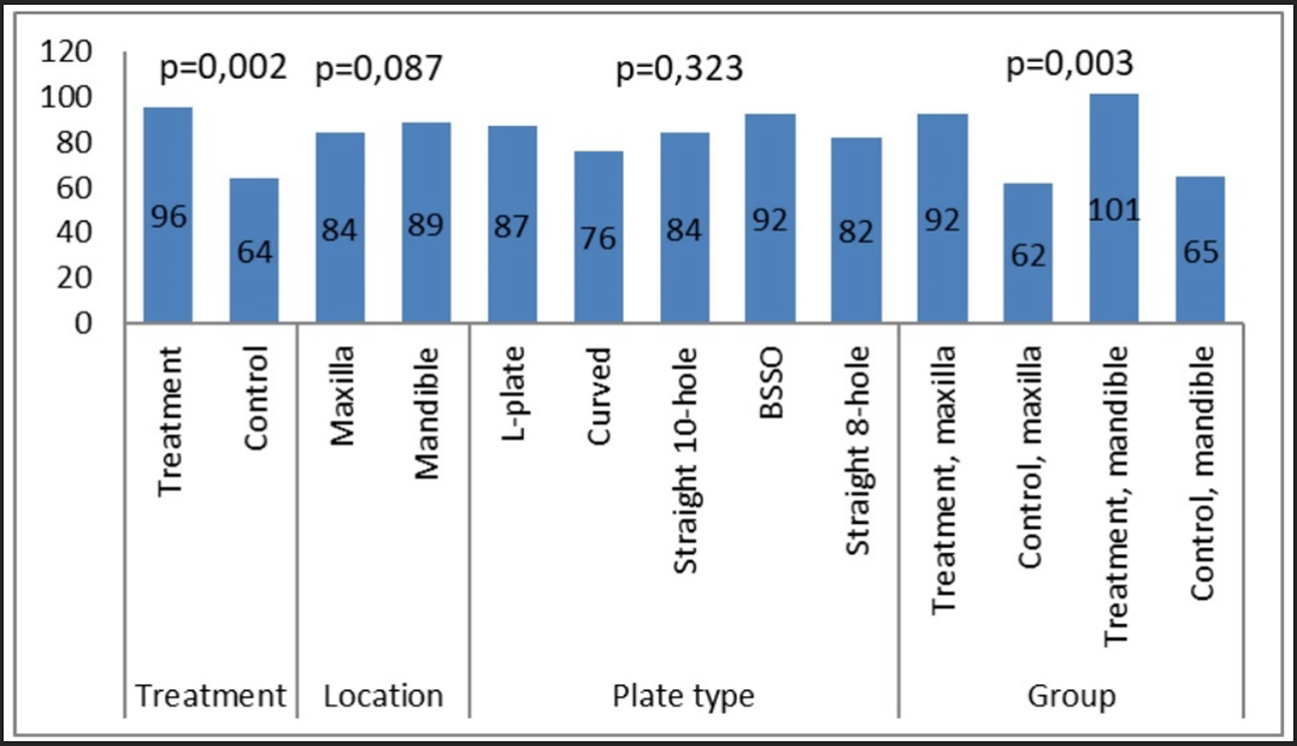
The results of the contact angle measurement on the collected plates and control plates.

The measurement of contact angle ( Figure 2) was conducted to evaluate the wettability and surface properties of the miniplates. The measurement was performed on all plates and thereafter compared according to their categories ( Figure 3). No notable changes in the contact angle were detected between the maxillary and mandibular plates, nor among the various miniplate types. There was a statistically significant difference in the contact angle between the treatment (patient-rejected) and control plates. The patient-rejected plates demonstrated a greater contact angle compared to the control plates. This result was constant when comparing the treatment and control groups for both maxillary and mandibular plates.

### Bacterial attachment

**Figure 4.**
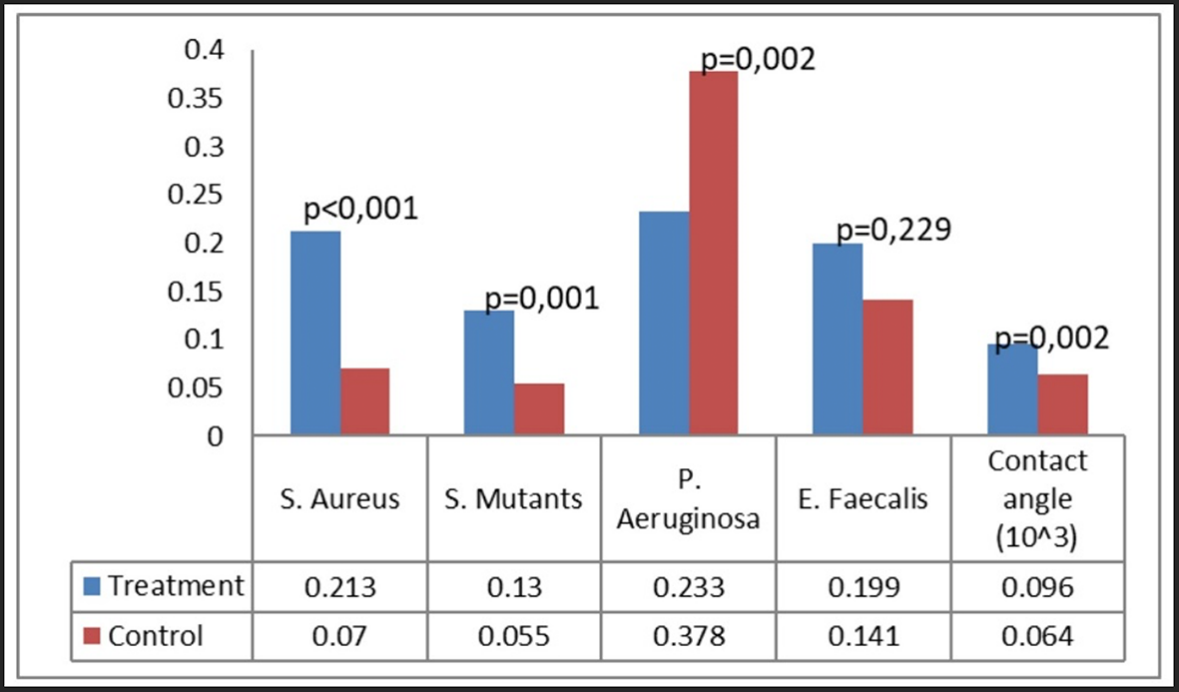
Bacterial attachment on patient-rejected plates compared to control (new plates).

Bacterial attachment was quantified utilizing the crystal violet technique, predicated on the optical density of the absorbed crystal violet. Four bacteria were examined: S. aureus, S. mutans, P. aeruginosa, and E. faecalis ( Figure 4). Our group noted certain patterns of bacterial adhesion on the miniplates. Miniplates rejected by patients shown markedly increased adhesion of S. aureus and S. mutans (P < 0.001), but P. aeruginosa exhibited greater adhesion on new plates relative to patient-rejected miniplates. The adhesion of E. faecalis was similar between patient-rejected and new plates.

**Figure 5.**
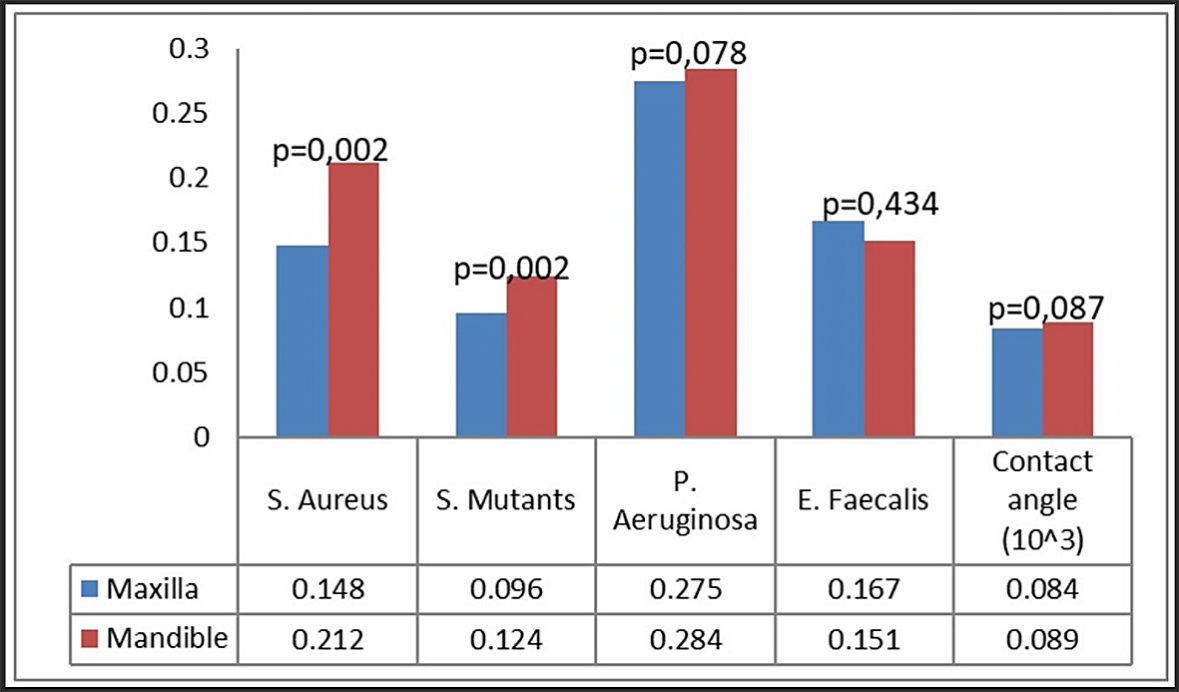
Bacterial attachment on maxillary and mandibular plates.

**Figure 6.**
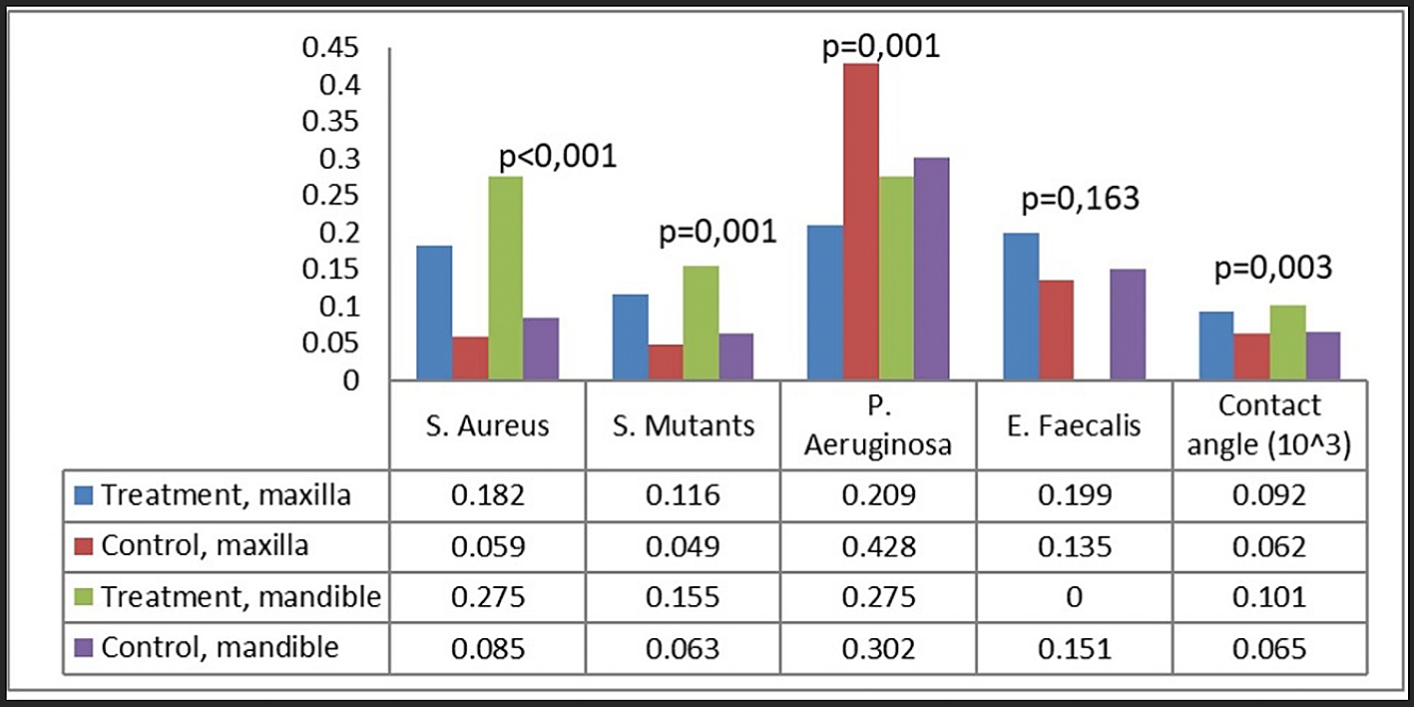
Bacterial attachment categorized as group and plate location.

In the comparison of maxillary and mandibular plates, S. aureus and S. mutans exhibited markedly more bacterial adherence on mandibular plates ( Figure 5). Both S. aureus and S. mutans demonstrated markedly increased adhesion on both maxillary and mandibular rejected plates ( Figure 6). No difference was found in the attachment of P. aeruginosa and E. faecalis between the maxillary and mandibular plates.

Analysis by plate location and treatment group revealed that the attachment of S. aureus and S. mutans was greatest on rejected mandibular plates, greatly surpassing that on control plates ( Figure 6). Rejected maxillary plates demonstrated markedly increased adhesion of S. aureus and S. mutans in comparison to control plates. The attachment pattern of P. aeruginosa differed from that of S. aureus and S. mutans, exhibiting more adhesion on maxillary control plates. The adhesion of E. faecalis was consistent across all groups.

**Figure 7.**
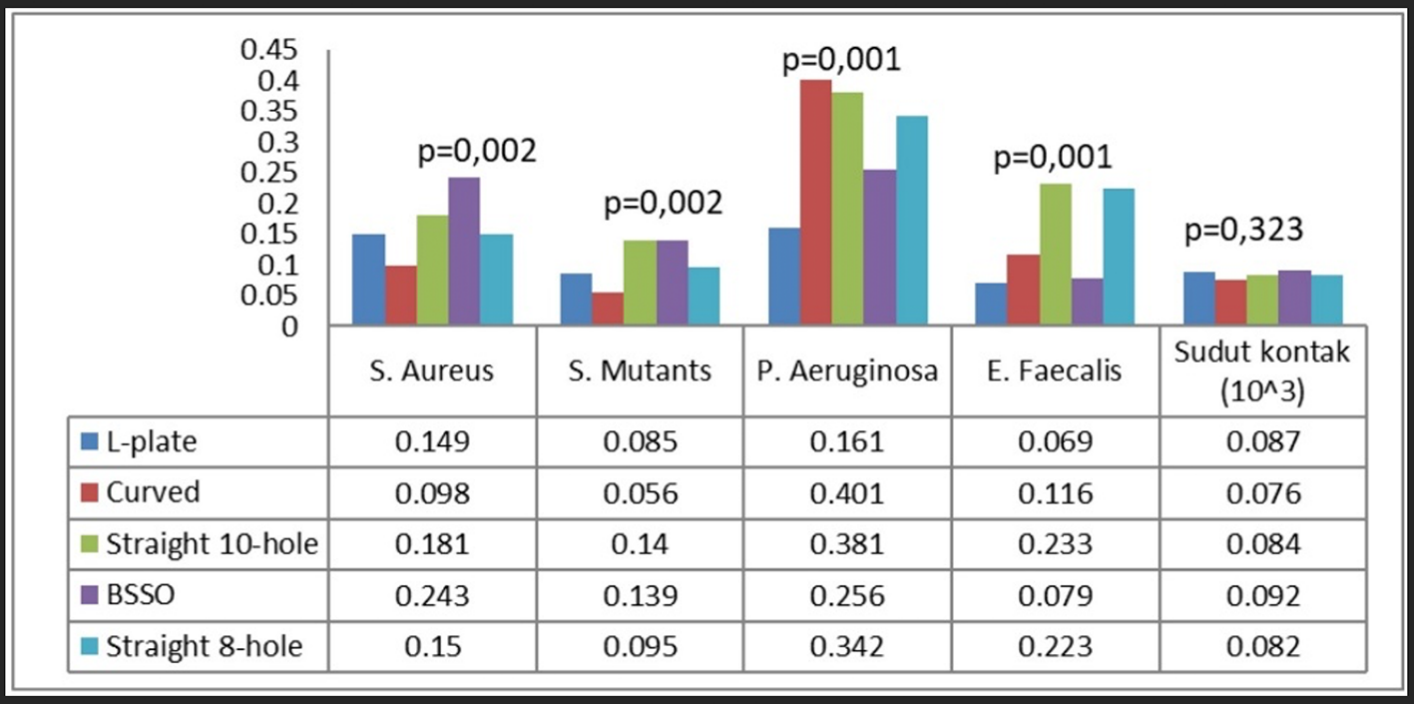
Bacterial attachment on different plate types.

Ultimately, each bacterium demonstrated distinct attachment patterns on different types of plates. S. aureus exhibited the greatest adhesion on BSSO straight-type plates, S. mutans on straight 10-hole and BSSO straight-type plates, P. aeruginosa on curved, straight 10-hole, and 8-hole plates, and E. faecalis on straight 10-hole and 8-hole plates ( Figure 7).

### SEM observation of bacterial attachment

SEM was utilized to examine the morphology and microstructural characteristics of bacterial adhesion on the miniplate surface. The adhesion of each bacteria was concurrently assessed on each miniplate, with specific emphasis on the bridge area owing to its constant bending and twisting.

**Figure 8.**
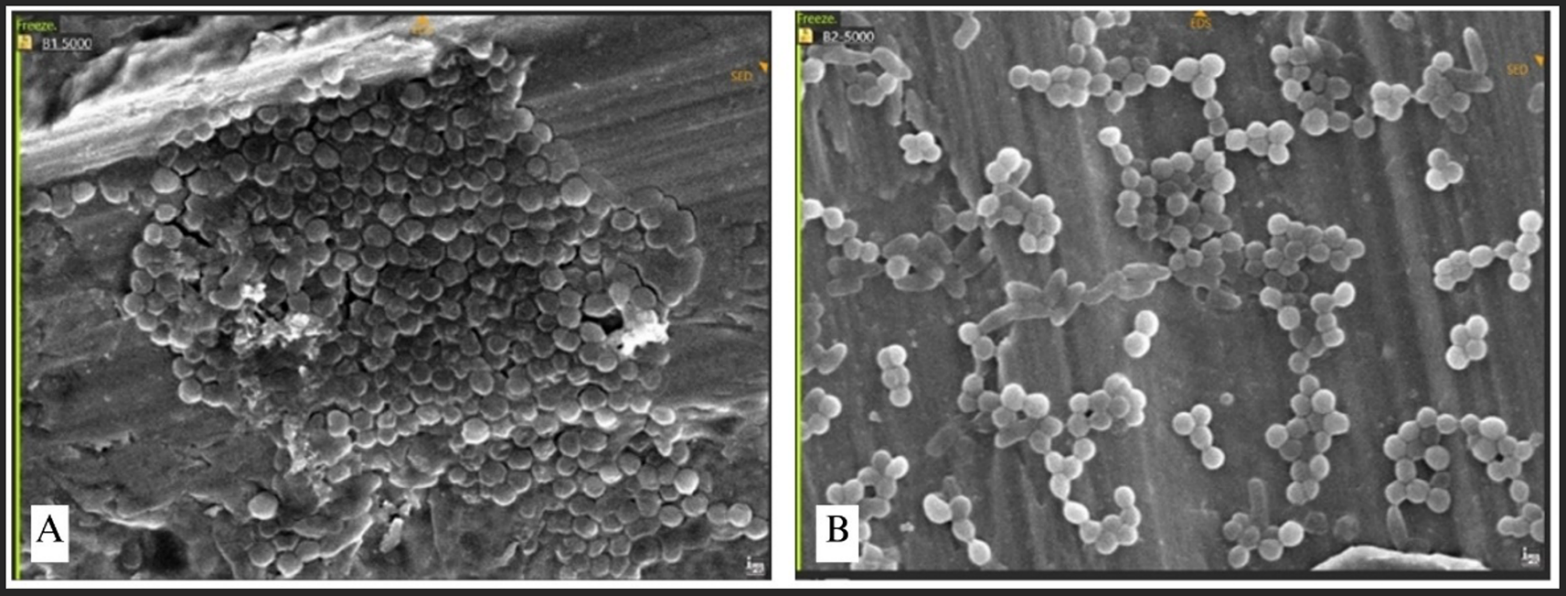
*Staphylococcus aureus* attachment observed by SEM, *Observation on* (A) L-shaped type miniplate, bridge area, 5000 × magnification, (B) Straight-type BSSO plate *,* bridge area, 5000 × magnification.

Bacterial adhesion was noted in all bridge regions, with each bacteria displaying unique attributes. Irregularities and porosity were seen in regions devoid of bacterial adhesion. The attachment of S. aureus manifested as clustered, high-density bacterial communities ( Figure 8). The morphology of S. aureus was distinctly visible as clustered cocci, adhering to the plate’s uneven surface. Bacterial adhesion on the straight-type BSSO plate was noted as uniformly distributed clusters with moderate density.

**Figure 9.**
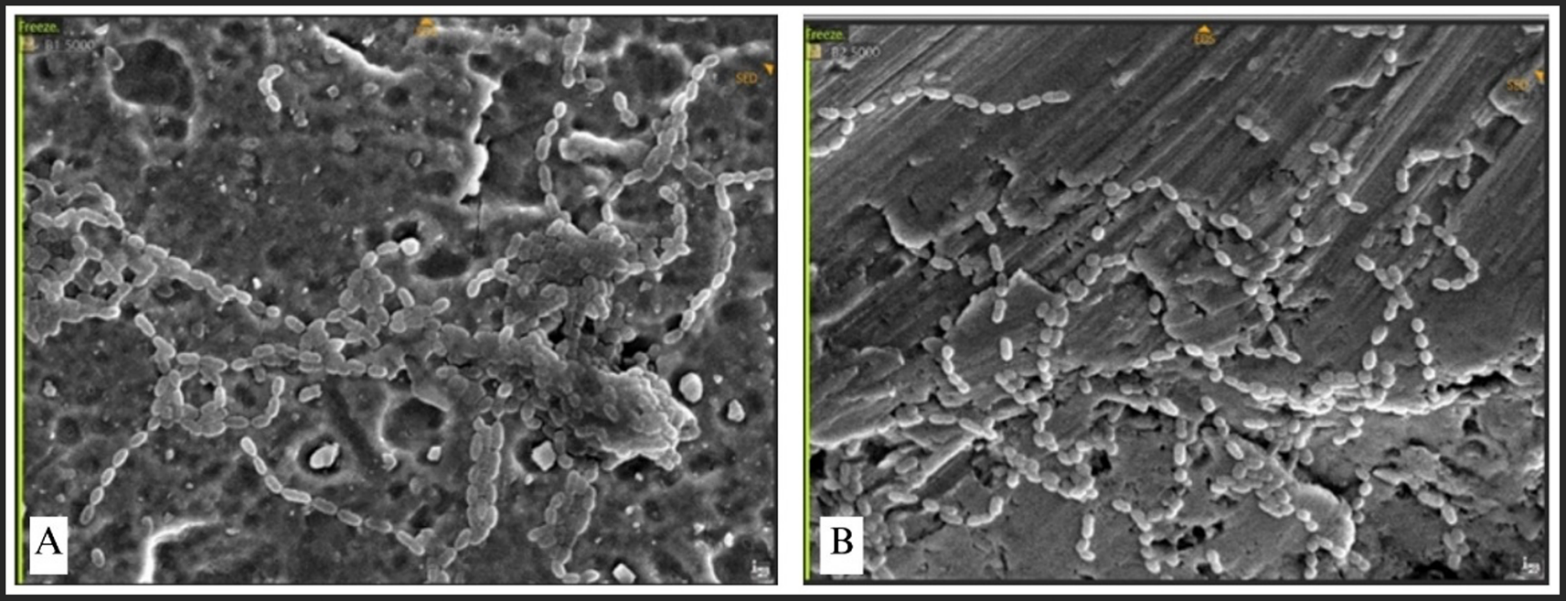
Streptococcus mutans attachment observed by SEM, Observation on (A) L-shaped type miniplate, bridge area, 5000 × magnification, (B) Straight-type BSSO plate, bridge area, 5000 × magnification.

**Figure 10.**
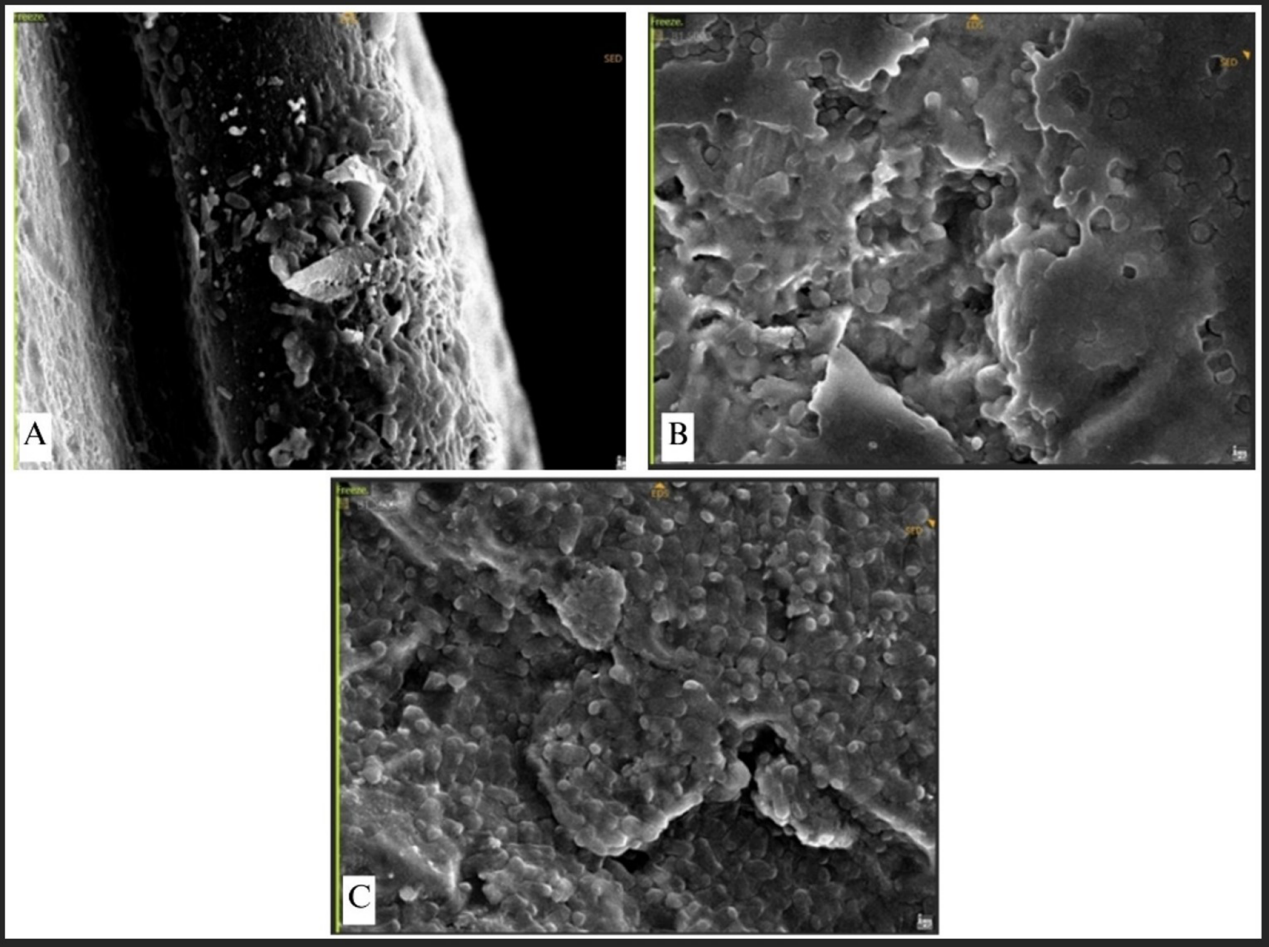
(A) *P. aeruginosa* attachment observed by SEM, Observation on L-shaped type miniplate, bridge area, 5000 × magnification, (B) *E. faecalis* attachment on Straight-type BSSO plate, bridge area, 5000 × magnification (C) *E. coli* attachment on Curve-type plate, bridge area, 5000 ×.

The attachment of Streptococcus mutans was marked by distinct chains of cocci ( Figure 9). The bacteria established a dense population, uniformly scattered around the plate. Conversely, P. aeruginosa, E. faecalis, and E. coli demonstrated exceptionally high-density bacterial populations, resulting in no discernible abnormalities on the surface of the plate ( Figure 10). This indicates that the bacterial biofilm encompassed the whole surface of the plate, reflecting a significant degree of bacterial adhesion.

## Discussion

This study examined osteosynthesis-associated infections (OAI) that require implant removal, potentially jeopardizing fracture stability and extending healing time, thereby elevating healthcare expenses. ^1^ Conventional interventions by oral and maxillofacial surgeons often encompass drainage and fracture stabilization with antibiotics; however, hardware extraction is required in instances of osteomyelitis or non-union of bone. ^6, 9^ Our data indicated a 2% incidence of OAI, consistent with the literature that cites infection rates between 2.7% to 26.8%. Infections associated with craniomaxillofacial (CMF) hardware occur less frequently than those related to extremities osteosynthesis, probably owing to the distinctive anatomy and robust blood supply of the facial region. Nonetheless, the mandible demonstrated markedly greater vulnerability to infection, as previously shown. ^1, 2, 10^

The most often found isolated bacteria were Streptococcus spp., Prevotella spp., and Staphylococcus spp., indicating their significant roles in oral wound infections. ^2, 11^ Pseudomonas aeruginosa was infrequently found, although it was considerably present in OAI cases associated with greater capacity plates. Infections caused by Streptococcus spp., Prevotella spp., and Staphylococcus spp. were more frequently linked to smaller volume plates, underscoring the intricate nature of osteoarticular infections, often facilitated by polymicrobial biofilms. This intricacy requires a comprehensive treatment strategy, encompassing antibiotics, debridement, wound management, and, in certain instances, hardware extraction. ^5, 12– 14^

The choice of bacterial strains (S. mutans ATCC 25175, P. aeruginosa, S. aureus ATCC 25933, and E. faecalis) for inoculation in infected miniplates fulfills several research objectives. The well-characterized reference strains, sourced from esteemed culture collections (ATCC), provide consistency and repeatability in experiments. ^15^ Each strain signifies a clinically relevant pathogen recognized for inducing infections in osteosynthesis, providing critical insights into infection processes and therapeutic approaches. ^15, 16^ The variety of these strains—encompassing both Gram-positive (e.g., S. mutans, S. aureus, E. faecalis) and Gram-negative (e.g., P. aeruginosa) bacteria—enables researchers to investigate different facets of biofilm development and antibiotic resistance. ^15, 17^ Moreover, employing several bacterial strains facilitates comparative analysis, enabling researchers to evaluate the effectiveness of treatments, such as antimicrobial drugs or surface coatings, against diverse pathogens, hence improving the relevance and consistency of research findings. ^15, 11^

Bacterial adherence to titanium miniplates differed, with S. aureus demonstrating the greatest attachment, succeeded by P. aeruginosa, E. faecalis, and S. mutans. The disparities may be ascribed to the mechanical properties and surface attributes of the miniplates. ^18^ Surface imperfections, like microfractures or irregularities, might foster conditions favorable for bacterial adherence. Staphylococcus aureus, recognized for its adhesive characteristics and biofilm formation abilities, presumably exploits these compromised surfaces, leading to increased colonization rates relative to other bacterial strains. The thickness of titanium miniplates may affect bacterial attachment, as thicker materials offer increased surface area and imperfections that facilitate bacterial colonization. Mechanical forces during implantation and subsequent motion might induce surface deformation, hence promoting bacterial adhesion. ^7, 19, 20^ Comprehending these pathways is crucial for formulating strategies to reduce bacterial colonization and biofilm development, hence enhancing outcomes in orthognathic surgery and facial reconstruction. ^21– 23^

The bending, twisting, and continuous loading of miniplates result in surface imperfections and degradation, facilitating bacterial adherence. The mechanical stresses, along with fluctuations in bone density in the mandible, lead to stress concentration and surface degradation, impacting the wettability and roughness of miniplates, both of which are directly associated with biofilm formation. Consequently, mandibular miniplates exposed to bending are expected to demonstrate increased hydrophilicity, offering insights into the mechanics of biofilm formation. ^7, 19, 23^

Anatomical variables additionally affect the correlation between surface damage and bacterial adhesion. The mandible’s intricate biomechanics and constant mobility complicate miniplate attachment, resulting in increased vulnerability to surface injury. The acute fracture angles frequently observed in mandibular fractures exacerbate the challenges of miniplate installation and heighten the potential of surface injury. Conversely, although bending pressures exert influence on maxillary miniplates, especially in buttress regions, surface degradation is less evident than in mandibular miniplates. These structural variations must be taken into account when evaluating infection susceptibility and formulating preventive strategies in orthognathic surgery and facial reconstruction. ^8^

The design of thicker miniplates in the mandible (1 mm) relative to the maxilla (0.5 mm) is noteworthy. Thicker miniplates provide an expanded surface area for bacterial colonization, which may result in enhanced biofilm formation. Moreover, bigger miniplates impose increased mechanical stress on adjacent tissues, perhaps leading to tissue damage or ischemia, so undermining the host’s immune response and facilitating biofilm development. The material composition and surface coatings of thicker miniplates affect bacterial adhesion and biofilm formation. ^24^

These variables underscore the significance of implant design in reducing the incidence of implant-associated infections. In result, our investigation elucidates the complicated interplay between bacterial colonization, implant volume, and osteosynthesis-related infections, providing significant insights for the management of these difficult clinical circumstances. ^25^

This research possesses multiple limitations. Initially, its in vitro design may not completely emulate the intricacies of in vivo surroundings, excluding elements such as immune response and tissue interactions. The absence of sample size and specifics of the studied miniplates may constrain statistical power. The study exclusively examined titanium miniplates, neglecting any variations in bacterial adhesion on alternative materials. It also lacks longitudinal follow-up to evaluate the evolution of bacterial populations over time. The research examined a restricted selection of bacterial strains and did not investigate differences in implant design, geometry, or coatings. Moreover, it failed to include clinical parameters, such as patient comorbidities or immunological status, that could affect infection outcomes. The influence of the host immunological response was not considered, nor were the impacts of surface imperfections and mechanical stresses comprehensively predicted. Ultimately, although bacterial attachment was noted, the growth and maturity of biofilms were not thoroughly investigated, constraining the comprehension of biofilm resistance to therapy.

## Conclusion

This study highlights the critical impact of surface damage on elevating the incidence of postoperative infections, attributable to increased bacterial attachment to titanium miniplates employed in osteosynthesis. Miniplates rejected by patients, particularly those exposed to mechanical stress, demonstrated increased bacterial adhesion, notably for S. aureus and S. mutans. The mandible exhibited increased bacterial colonization due to its intricate biomechanics and surface imperfections, whereas the maxillary plates shown reduced susceptibility. These findings underscore the necessity of preserving the integrity of miniplates during and post-orthognathic surgery to mitigate infection risk. The research underscores the necessity of meticulously evaluating implant design, material characteristics, and anatomical considerations to avert implant-associated infections. Additional study is crucial to formulate effective preventive and management methods for infections linked to miniplate surface degradation and to enhance clinical results in osteosynthesis.

## Ethics approval

This study was approved by the Ethics Committee of the Faculty of Dentistry – Prof. Soedomo Dental Hospital, Universitas Gadjah Mada on July 16, 2024, with a number: 150/UN1/KEP/FKG-RSGM/EC/2024. In addition, this study adheres to the Declaration of Helsinki ( https://www.wma.net/policies-post/wma-declaration-of-helsinki-ethical-principles-for-medical-research-involving-human-subjects/).

## CRediT authorship contribution statement

Conceived and designed the experiments: BPS TH. Analyzed the data: BPS AK. Wrote the paper: BPS TH. Designed search strategies: BPS AK TH. Critically reviewed the manuscript for important intellectual content: BPS AK MGW TH. Read and approved the final version: BPS AK MGW TH. Guarantors: BPS TH.

## Consent

Informed written consent was acquired from all individual participants or their guardians if they are children. Furthermore, valid informed consent was acquired from all individual participants for the publication of their data.

## Data Availability

Raw underlying data: https://doi.org/10.5281/zenodo.14728347. ^26^ Data are available under the terms of the Creative Commons Attribution 4.0 International license (CC-BY 4.0). Reporting guidelines, STROBE checklist: https://doi.org/10.5281/zenodo.14610150. ^27^ Data are available under the terms of the Creative Commons Attribution 4.0 International license (CC-BY 4.0).
