# The Influence of Surface Damage on Miniplates: A Study of Bacterial Attachment Across Various Strains

**DOI:** 10.12688/f1000research.159954.2

**Published:** 2025-05-21

**Authors:** Bramasto Purbo Sejati, Tetiana Haniastuti, Ahmad Kusumaatmaja, Maria Goreti Widyastuti

**Affiliations:** 1 Departemnt of Oral and maxillofacial Surgery, Universitas Gadjah Mada, Yogyakarta, Special Region of Yogyakarta, Indonesia; 2 Department of Oral Biology, Universitas Gadjah Mada, Yogyakarta, Special Region of Yogyakarta, Indonesia; 3 Department of Physics, Faculty of Mathematics and Natural Sciences, Universitas Gadjah Mada, Yogyakarta, Special Region of Yogyakarta, Indonesia

**Keywords:** surface damage, bacterial attachment, contact angle

## Abstract

**Background:**

Postoperative infection and rejection of miniplates in maxillofacial surgery are linked to surface irregularities and bacterial adhesion. This study investigated the physical and microbiological characteristics of patient-rejected miniplates to elucidate factors contributing to implant failure.

**Methods:**

Forty miniplates, including straight-type BSSO and L-shaped designs, were collected from patients. Macro photography documented surface deformities. Contact angle measurements assessed surface wettability. Bacterial adhesion for Staphylococcus aureus, Streptococcus mutans, Pseudomonas aeruginosa, and Enterococcus faecalis was quantified via the crystal violet assay. Scanning electron microscopy (SEM) was used to visualize microbial colonization.

**Results:**

Macro images showed visible deformations, especially in the bridge areas of straight-type BSSO plates. Contact angle analysis revealed significantly greater hydrophobicity in rejected plates than controls (mean: 89.6° vs. 72.3°, p < 0.01). Rejected plates demonstrated significantly increased adhesion of S. aureus and S. mutans (p < 0.001), particularly on mandibular plates. P. aeruginosa adhered more to control plates (p < 0.05), while E. faecalis showed no significant difference between groups. SEM confirmed dense bacterial clusters, with S. aureus forming cocci aggregates and S. mutans forming chains, particularly in regions of surface distortion.

**Conclusion:**

Rejected miniplates exhibit increased surface roughness and hydrophobicity, correlating with elevated bacterial adhesion. These findings highlight the need for improved surface design or coating technologies to reduce biofilm formation and enhance clinical outcomes in maxillofacial surgery.

## Introduction

Postoperative infections remain a significant complication in maxillofacial surgery, ^1^ particularly when osteosynthesis miniplates are employed for fracture stabilization. Titanium is widely used due to its biocompatibility and mechanical reliability; however, intraoperative manipulation such as bending may alter its surface microstructure, potentially enhancing bacterial colonization and biofilm formation. ^1– 3^

Biofilms present a major challenge in osteosynthesis, as they confer resistance to antibiotics and host immune responses. ^3, 4^ These infections often necessitate premature implant removal, leading to prolonged healing, chronic inflammation, and increased healthcare costs. ^4– 6^ Despite prophylactic antibiotic use, infection rates following osteosynthesis range from 5–14%, varying with anatomical location, fracture complexity, and patient-specific risk factors. ^6– 8^ For instance, patients with poor periodontal health or intraoral plate placement are significantly more prone to infection. ^8– 10^

Recent literature suggests that a multifactorial approach is essential for preventing biofilm-related complications. This includes not only prophylactic antibiotics but also emerging techniques such as antimicrobial coatings, patient-specific implants, and novel biomaterials like PEEK, which have shown reduced bacterial adherence. ^10– 13^ Nevertheless, the specific impact of mechanical surface damage—such as that incurred during intraoperative bending of titanium plates—on microbial adhesion and biofilm formation remains poorly understood. ^13, 14^

Bicsák et al. reported that three-dimensional adaptation of titanium plates does not increase complication rates, but their study lacked microbiological and surface-level data. ^14, 15^ Conversely, Knabl et al. found that over 50% of explanted osteosynthesis materials, even from asymptomatic patients, were colonized by bacteria, underscoring the silent progression of implant-associated infections. ^15, 16^ Furthermore, complex interspecies biofilm interactions—such as those involving *Staphylococcus aureus*, *Streptococcus mutans*, *Enterococcus faecalis*, and *Pseudomonas aeruginosa*—can heighten resistance and pathogenicity, making early surface-level interventions critical. ^16, 17^

This study aims to investigate the impact of surface modifications on bacterial attachment to osteosynthetic miniplates. Specifically, titanium miniplates retrieved from patients due to infection or rejection will be analyzed to understand the role of clinically induced surface alterations in promoting microbial colonization. The study will focus on characterizing surface properties—such as hydrophobicity, roughness, and topographical changes—using scanning electron microscopy (SEM), atomic force microscopy (AFM), and contact angle measurements. Subsequently, these miniplates will be exposed to four clinically relevant bacterial strains ( *Staphylococcus aureus*, *Streptococcus mutans*, *Enterococcus faecalis*, and *Pseudomonas aeruginosa*) to assess species-specific patterns of adhesion and biofilm formation. Quantitative analysis of bacterial colonization and the spatial distribution of microorganisms on damaged versus undamaged plate surfaces will be conducted to elucidate correlations between surface characteristics and microbial attachment.

## Methods

This study employed a retrospective observational design, analyzing titanium miniplates removed from patients treated at Temanggung Regional Hospital between 2020 and 2023 due to clinical signs of infection. This study adheres to the STROBE reporting guideline. This study’s inclusion criteria mandated that patients possess simple fractures treated with titanium alloy miniplates from the Osteomed system, specifically 1.6 mm and 2.0 mm models from Acumed, USA. The miniplates were required to be non-locking or adaptation plates, with or without extended plates, in the maxillofacial region, and must have undergone adaptation through bending, with or without twisting. The miniplates must have been implanted for over two weeks and demonstrate clinical signs of infection, including exposed miniplates, pus in the surrounding area, elevated leukocyte counts, and non-union evident in radiological imaging. Miniplates that could be easily removed without substantial difficulty and without necessitating burring of the bone were also included.

The exclusion criteria removed patients under 17 years of age or over 65 years, individuals with infections associated with medically compromised conditions, patients with comminuted, infected, or multiple fractures, and those whose surgical procedures exceeded two hours in duration. Additionally, patients who did not comply with postoperative instructions, especially concerning antibiotic use, were excluded.

According to the established criteria, 12 infected miniplates were identified from a total of 651 miniplates implanted throughout the study period. A total of 12 miniplates were collected from 10 patients among 492 treated with miniplates. The study involved ten adaptation-type Osteomed miniplates: seven 1.6 mm miniplates (consisting of two four-hole L-shaped miniplates, two five-hole curved miniplates, and three four-hole straight miniplates) and three 2.0 mm miniplates of the four-hole extended type.

This study was approved by the Ethics Committee of the Faculty of Dentistry – Prof. Soedomo Dental Hospital, Universitas Gadjah Mada on July 16, 2024 with a number: 150/UN1/KEP/FKG-RSGM/EC/2024. In addition, this study adhere to the Declaration of Helsinki ( https://www.wma.net/policies-post/wma-declaration-of-helsinki-ethical-principles-for-medical-research-involving-human-subjects/).

### Miniplate retrieval and handling

Following surgical removal due to infection or rejection, each miniplate was immediately placed into a sterile container without any tissue preservation medium. The samples were transported to the laboratory under sterile conditions. Prior to macroscopic imaging and bacterial culture procedures, all miniplates were sterilized using an autoclave to prevent cross-contamination between specimens.

### Macro photography of miniplates using a camera

The macro photography process utilizes a DSLR or mirrorless camera with a macro lens, exemplified by the Nikon Z50 paired with the Nikon AFS 60mm f/2.8 G Micro-Nikkor lens. Diffused natural or artificial lighting was configured using a minimum of two light sources positioned at 45-degree angles to the miniplate to achieve uniform illumination and reduce shadows. Camera settings were optimized with an aperture range of f/8 to f/11 to achieve adequate depth of field, a shutter speed between 1/125 and 1/250 seconds for precise exposure, and an ISO setting of 100 to 400 to ensure optimal clarity.

The miniplate was precisely placed within the camera’s viewfinder or LCD screen, occupying around 70% of the frame to achieve optimal composition. A minimum of 10 photographs were captured from multiple perspectives, including top-down, side, and angled views, to ensure thorough documentation. Images were examined on a computer monitor at 100% magnification, and the clearest, most detailed photographs were chosen for analysis. Surface texture, defects, and dimensions were meticulously documented for comprehensive record-keeping.

### Assessment of contact angle on miniplates

The contact angle serves as a crucial parameter for evaluating wettability and surface properties. Measurement is conducted using specialized instruments, including a contact angle goniometer. The angle formed at the liquid-solid interface tangent to the miniplate surface reflects the liquid’s spreading and adhesion characteristics. A 3 μl droplet of liquid was deposited onto the miniplate surface, and the droplet profile image was recorded using a custom device linked to a digital camera. Two formulas were utilized to determine the contact angle from the drop profile image: the linear gradient equation and the tangential line method.

Macro photography was employed to visually document deformation and irregularities on the miniplate surfaces, quantitative surface roughness measurements using profilometry or atomic force microscopy (AFM) were not performed. This limitation was due to the complex, patient-specific shapes of the miniplates following clinical adaptation via bending and twisting to fit facial anatomy. As a result, the plate surfaces were no longer flat, which posed significant challenges for obtaining consistent and accurate roughness data—a prerequisite for both profilometric scanning and AFM imaging.

### Bacterial attachment assessment

*Preparation of microorganisms and inoculum*

Strains of S. mutans (ATCC 25175), P. aeruginosa, S. aureus (ATCC 25933), and E. faecalis were obtained from the Integrated Research Laboratory at the Faculty of Dentistry, Universitas Gadjah Mada, Indonesia. A single colony of S. mutans, P. aeruginosa, S. aureus, or E. faecalis was cultured in BHI broth medium at 37°C for 24 hours. The turbidity of the bacterial suspension was adjusted to 0.5 McFarland, corresponding to 1.5 × 10 ^8^ CFU/mL. One milliliter of the bacterial suspension was introduced into 9 mL of BHI broth (1.10493.0500, Merck, Germany) medium within a Petri dish. The miniplate was immersed in the culture medium and incubated at 37°C for durations of 24 and 48 hours. Phosphate-buffered saline (PBS) was employed to rinse the adhered biofilm. The biofilm was stained with 0.1% crystal violet for 15 minutes, followed by washing with PBS, and subsequently treated with 96% ethanol to elute the bound crystal violet. The absorbance of the released crystal violet in ethanol was quantified at OD540 nm utilizing a spectrophotometer (Thermo Scientific, USA).

### Miniplate imaging utilizing Scanning Electron Microscopy (SEM)

Twelve infected miniplate samples and five unused control plates were analyzed using a Quanta 200 SEM (FEI, Oregon, USA). The plates were thoroughly dried prior to imaging due to the high vacuum conditions of the SEM. The plates were first removed from their containers, followed by the drainage of formalin and rinsing in buffer. Dehydration was conducted using a graded ethanol series, with each concentration (30%, 50%, 70%, 95%, and absolute ethanol) applied for 15 minutes, followed by three rinses in absolute ethanol. The plates underwent critical point drying utilizing a CPD 030 Critical Point Drier (Balzer, Leica, Solms, Germany) to reduce the potential for damage to fragile organic material. The plates were mounted onto aluminum SEM stubs with carbon tabs (Agar Scientific, Stansted, England) and subsequently sputter-coated with gold-palladium utilizing a Polaron E5100 SEM Coating Unit (Quorum Technology, East Grinstead, England) before imaging. The examination of plate and screw surfaces concentrated on pinpointing regions susceptible to biofilm formation, including surface protrusions, scratches, screw threads, screw hole depressions, and blood clots. Each patient sample necessitated approximately 5 hours for systematic evaluation and documentation based on the scanned photographic images.

### Control plate specification

The control plates used in this study were unused, sterile titanium miniplates obtained directly from the same manufacturer (Osteomed, Acumed, USA) and of the same type and dimensions as those used clinically (1.6 mm and 2.0 mm adaptation miniplates). These plates had never been implanted or exposed to any external environment and were handled under aseptic conditions throughout the experimental procedures.

### Statistical analysis

The biofilm formation inhibition assay was replicated independently a minimum of five times. The results are expressed as the mean ± standard deviation (SD) from one representative experiment. Since the data is not normally distributed, the Kruskal-Wallis test, followed by a post hoc Mann-Whitney test, was performed to evaluate the significance between groups. Statistical analysis utilized SPSS software, version 16.0, with a significance threshold established at p < 0.05.

## Result

### Macro photography of miniplates

**Figure 1.**
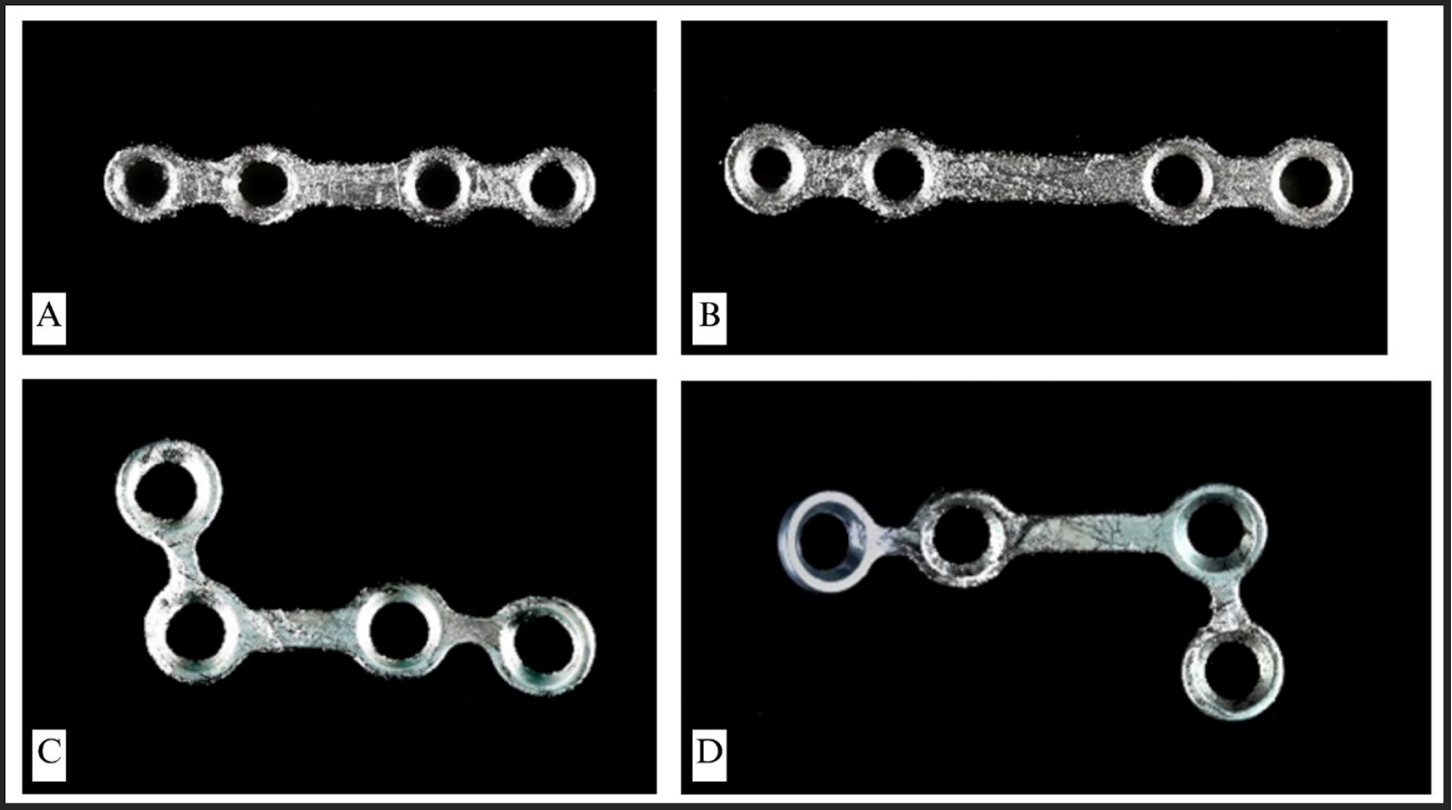
Macro photograph of the rejected miniplates. (A) and (B) Straight-type BSSO miniplates, (C) and (D) L-shaped type miniplates.

A macro image of the infected or rejected miniplates is presented ( Figure 1). A variety of plates were gathered, comprising straight-type BSSO plates and L-shaped plates. All collected plates displayed discernible surface irregularities and abnormalities. The bridge section of the straight-type BSSO plates exhibited the greatest deformation, presumably due to recurrent bending and twisting in this region. L-shaped plates had evident deformities and surface irregularities, with the most prominent distortion occurring on two sides of the plates.

### Calculation of contact angle on miniplates

**Figure 2.**
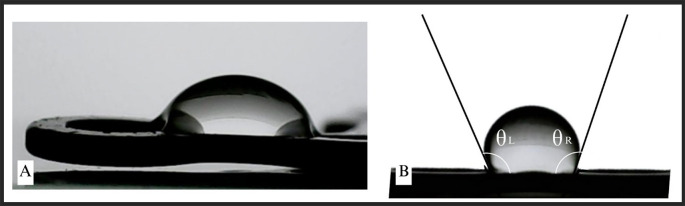
Contact angle measurement on the miniplates.

**Figure 3.**
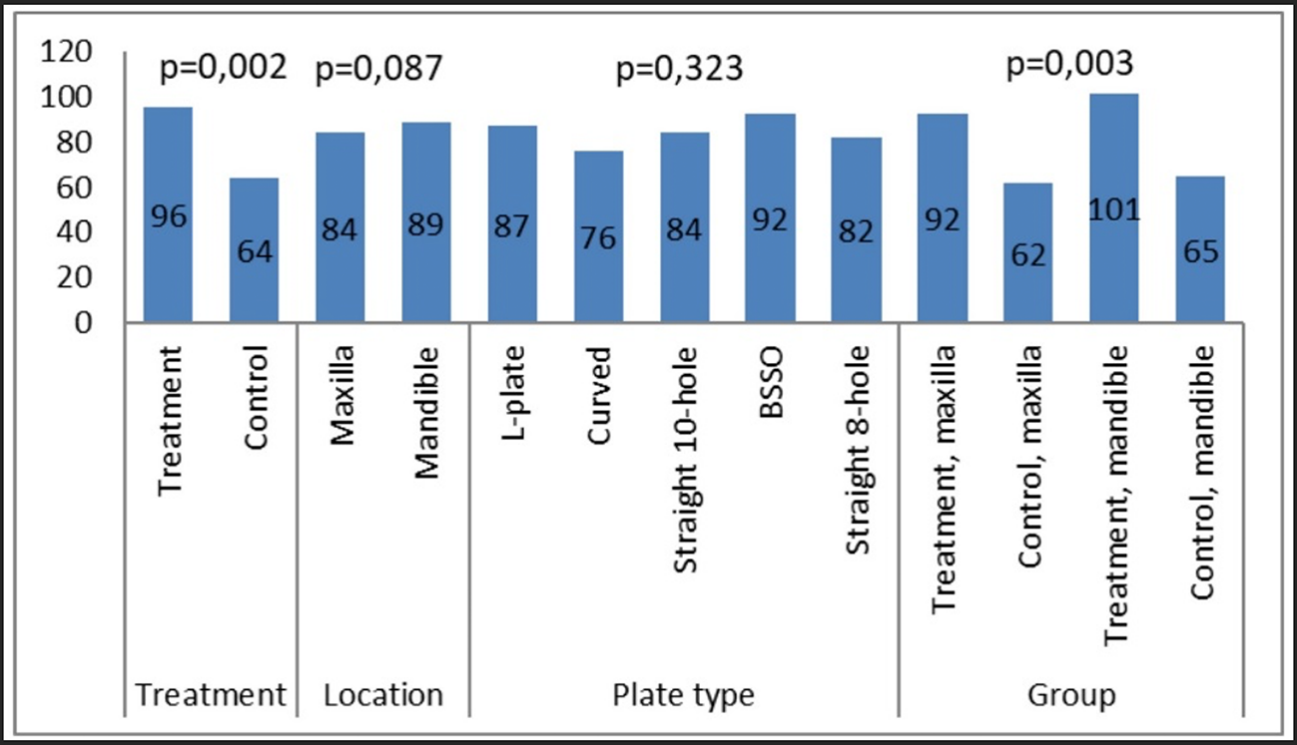
The results of the contact angle measurement on the collected plates and control plates.

The measurement of contact angle ( Figure 2) was conducted to evaluate the wettability and surface properties of the miniplates. The measurement was performed on all plates and thereafter compared according to their categories ( Figure 3). No notable changes in the contact angle were detected between the maxillary and mandibular plates, nor among the various miniplate types. There was a statistically significant difference in the contact angle between the treatment (patient-rejected) and control plates. The patient-rejected plates demonstrated a greater contact angle compared to the control plates. This result was constant when comparing the treatment and control groups for both maxillary and mandibular plates.

### Bacterial attachment

**Figure 4.**
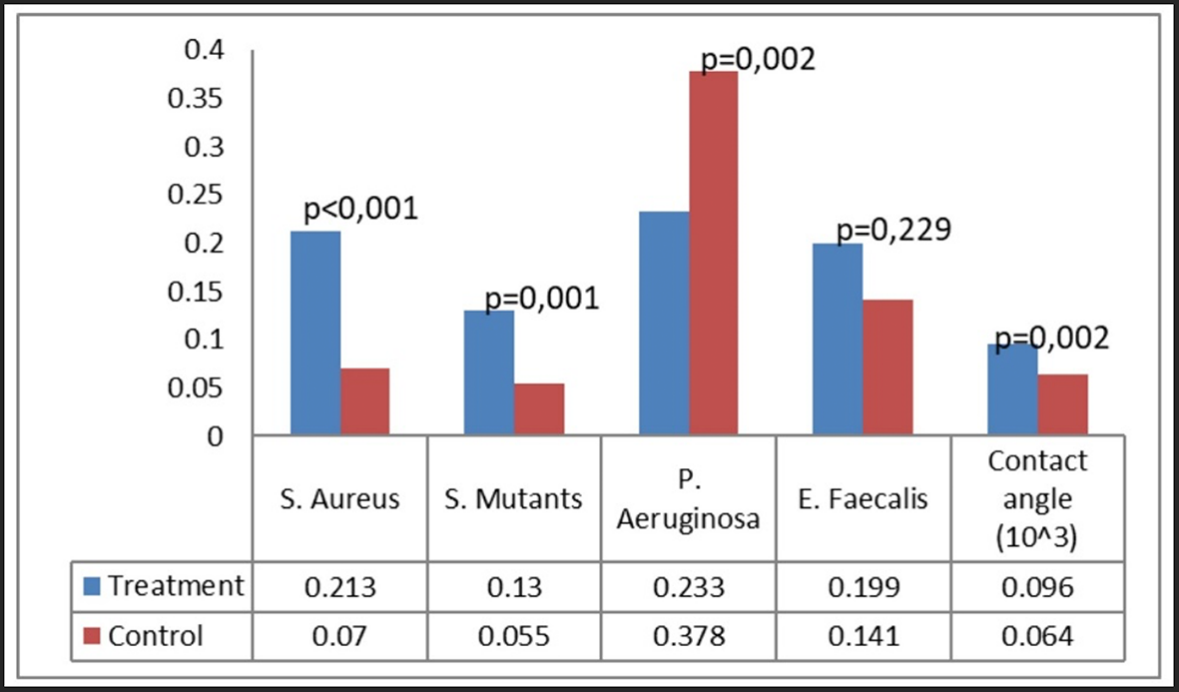
Bacterial attachment on patient-rejected plates compared to control (new plates).

Bacterial attachment was quantified utilizing the crystal violet technique, predicated on the optical density of the absorbed crystal violet. Four bacteria were examined: S. aureus, S. mutans, P. aeruginosa, and E. faecalis ( Figure 4). Our group noted certain patterns of bacterial adhesion on the miniplates. Miniplates rejected by patients shown markedly increased adhesion of S. aureus and S. mutans (P < 0.001), but P. aeruginosa exhibited greater adhesion on new plates relative to patient-rejected miniplates. The adhesion of E. faecalis was similar between patient-rejected and new plates.

**Figure 5.**
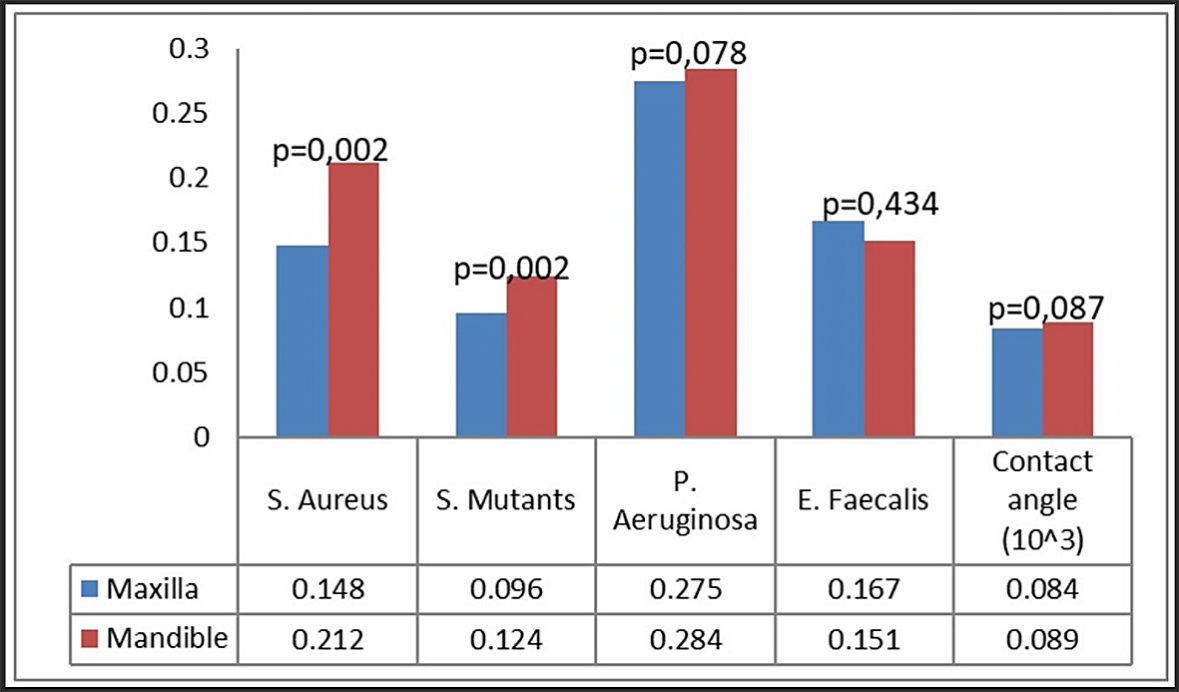
Bacterial attachment on maxillary and mandibular plates.

**Figure 6.**
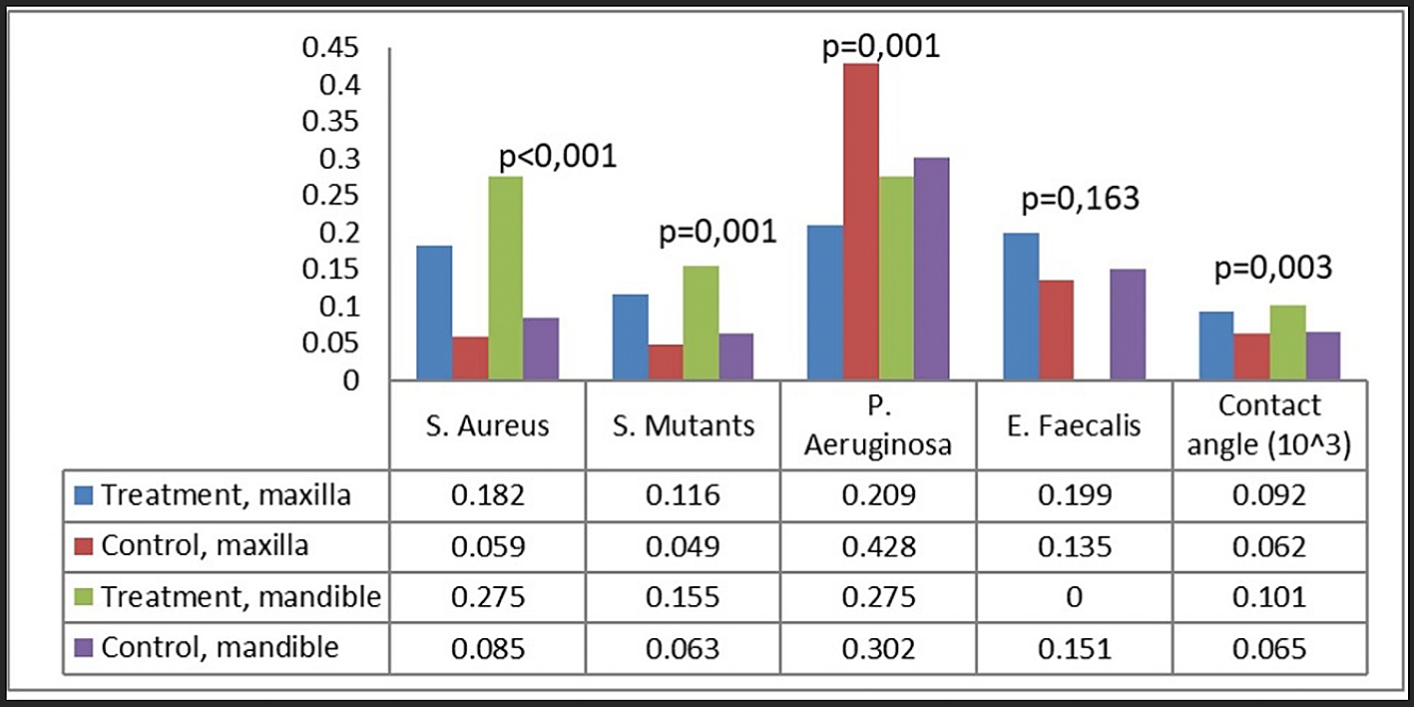
Bacterial attachment categorized as group and plate location.

In the comparison of maxillary and mandibular plates, S. aureus and S. mutans exhibited markedly more bacterial adherence on mandibular plates ( Figure 5). Both S. aureus and S. mutans demonstrated markedly increased adhesion on both maxillary and mandibular rejected plates ( Figure 6). No difference was found in the attachment of P. aeruginosa and E. faecalis between the maxillary and mandibular plates.

Analysis by plate location and treatment group revealed that the attachment of S. aureus and S. mutans was greatest on rejected mandibular plates, greatly surpassing that on control plates ( Figure 6). Rejected maxillary plates demonstrated markedly increased adhesion of S. aureus and S. mutans in comparison to control plates. The attachment pattern of P. aeruginosa differed from that of S. aureus and S. mutans, exhibiting more adhesion on maxillary control plates. The adhesion of E. faecalis was consistent across all groups.

**Figure 7.**
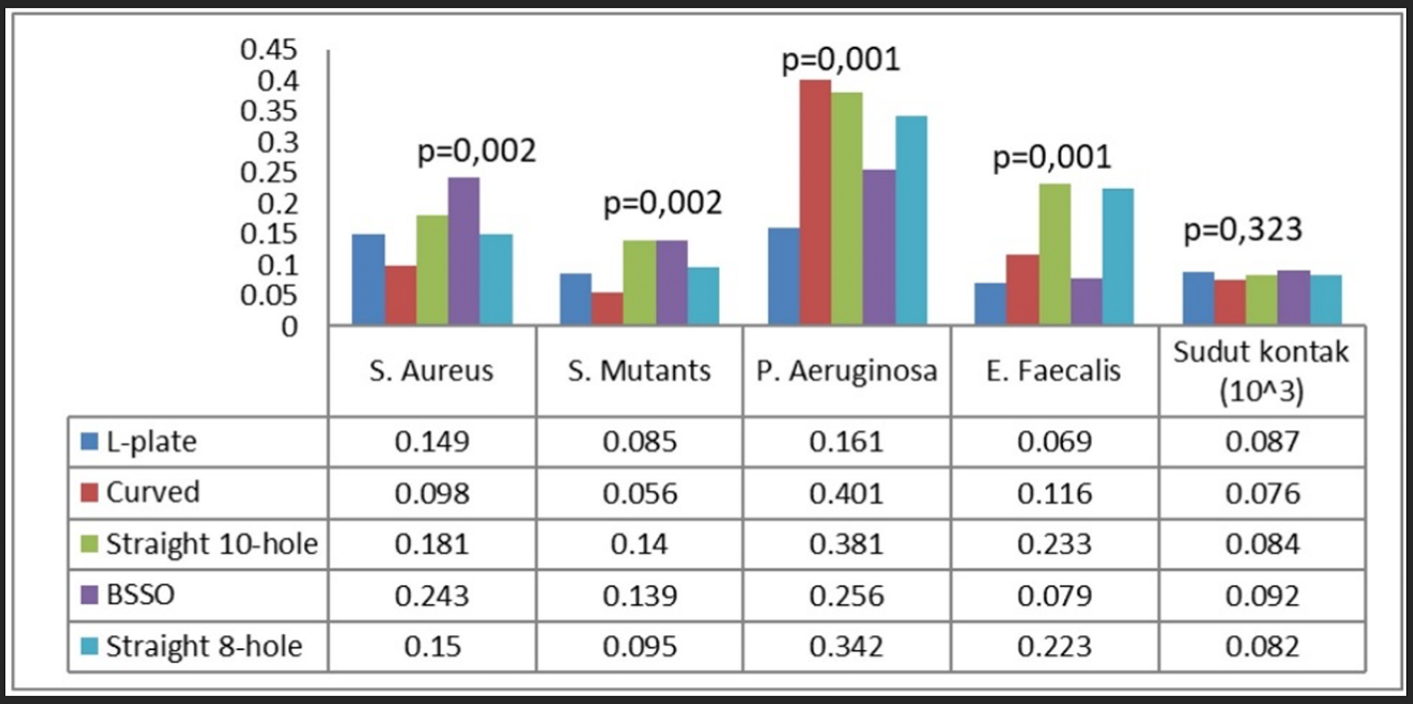
Bacterial attachment on different plate types.

Ultimately, each bacterium demonstrated distinct attachment patterns on different types of plates. S. aureus exhibited the greatest adhesion on BSSO straight-type plates, S. mutans on straight 10-hole and BSSO straight-type plates, P. aeruginosa on curved, straight 10-hole, and 8-hole plates, and E. faecalis on straight 10-hole and 8-hole plates ( Figure 7).

### SEM observation of bacterial attachment

SEM was utilized to examine the morphology and microstructural characteristics of bacterial adhesion on the miniplate surface. The adhesion of each bacteria was concurrently assessed on each miniplate, with specific emphasis on the bridge area owing to its constant bending and twisting.

**Figure 8.**
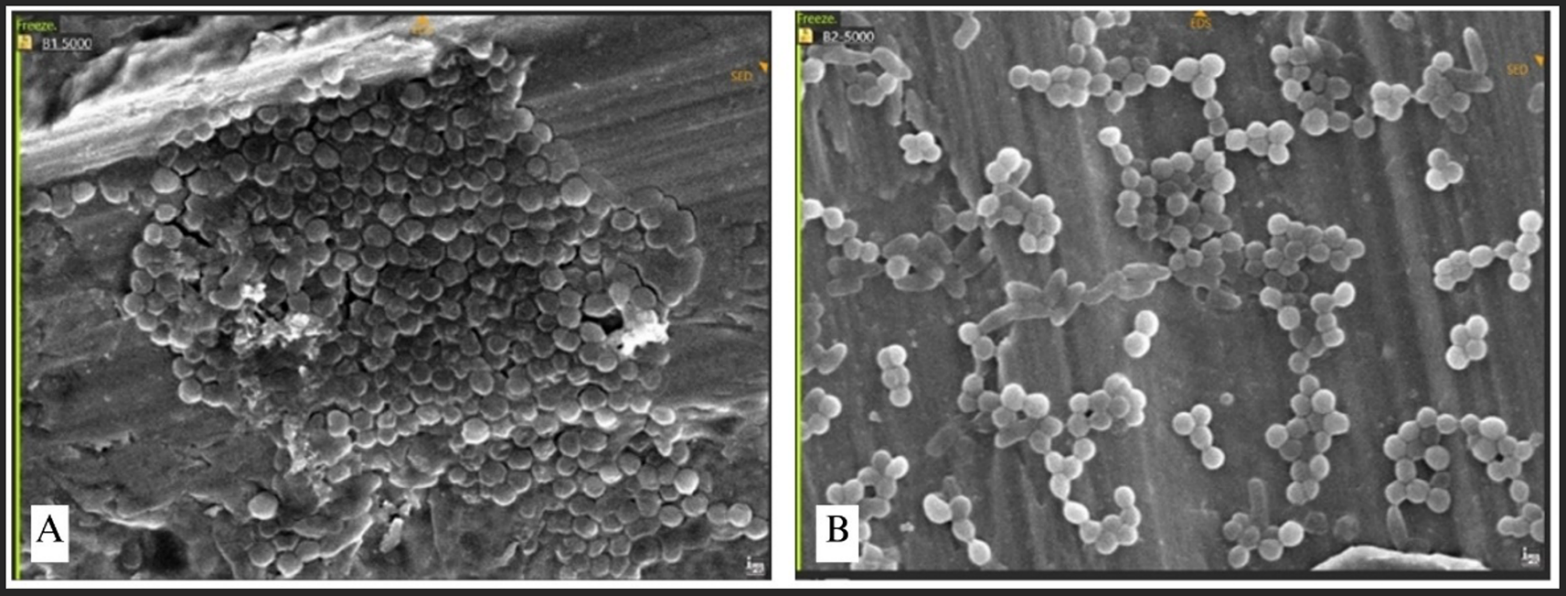
*Staphylococcus aureus* attachment observed by SEM, *Observation on* (A) L-shaped type miniplate, bridge area, 5000 × magnification, (B) Straight-type BSSO plate *,* bridge area, 5000 × magnification.

Bacterial adhesion was noted in all bridge regions, with each bacteria displaying unique attributes. Irregularities and porosity were seen in regions devoid of bacterial adhesion. The attachment of S. aureus manifested as clustered, high-density bacterial communities ( Figure 8). The morphology of S. aureus was distinctly visible as clustered cocci, adhering to the plate’s uneven surface. Bacterial adhesion on the straight-type BSSO plate was noted as uniformly distributed clusters with moderate density.

**Figure 9.**
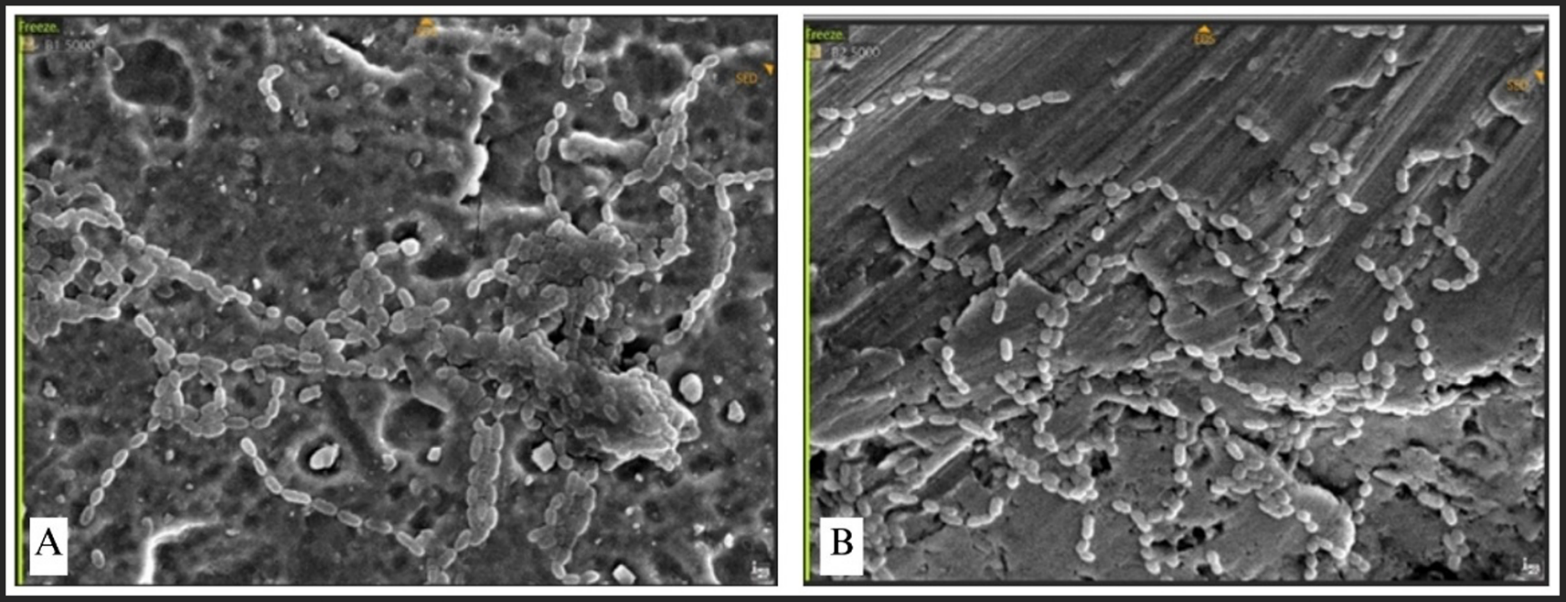
Streptococcus mutans attachment observed by SEM, Observation on (A) L-shaped type miniplate, bridge area, 5000 × magnification, (B) Straight-type BSSO plate, bridge area, 5000 × magnification.

**Figure 10.**
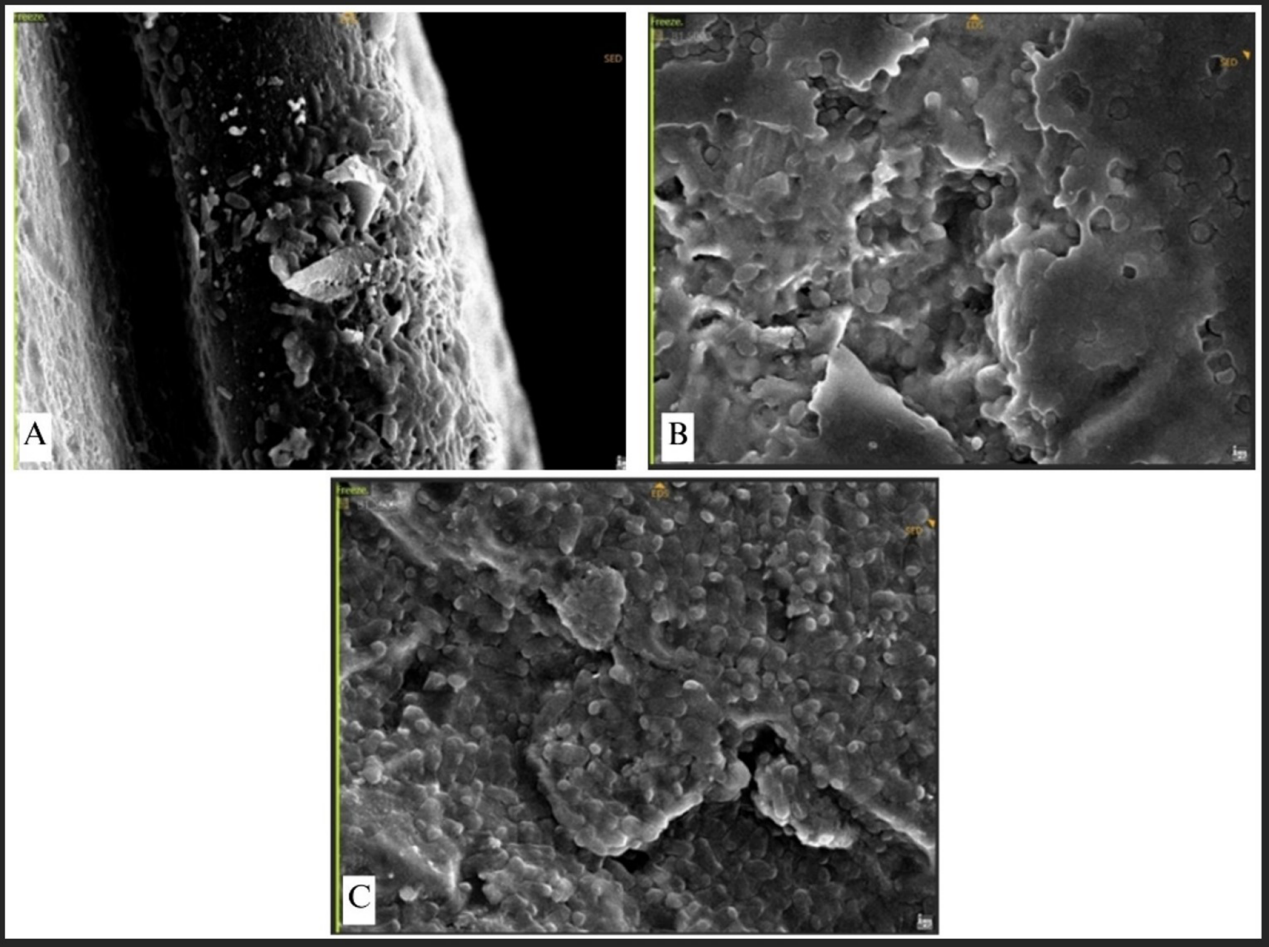
(A) *P. aeruginosa* attachment observed by SEM, Observation on L-shaped type miniplate, bridge area, 5000 × magnification, (B) *E. faecalis* attachment on Straight-type BSSO plate, bridge area, 5000 × magnification (C) *E. coli* attachment on Curve-type plate, bridge area, 5000 ×.

The attachment of Streptococcus mutans was marked by distinct chains of cocci ( Figure 9). The bacteria established a dense population, uniformly scattered around the plate. Conversely, P. aeruginosa, E. faecalis, and E. coli demonstrated exceptionally high-density bacterial populations, resulting in no discernible abnormalities on the surface of the plate ( Figure 10). This indicates that the bacterial biofilm encompassed the whole surface of the plate, reflecting a significant degree of bacterial adhesion.

### Wound dehiscence and exposure to oral secretions

In several cases included in this study, local wound dehiscence was observed, contributing to the clinical exposure of miniplates to the oral environment. Although primary wound healing was initially achieved in most patients, factors such as mechanical irritation, poor oral hygiene, infection, and soft tissue tension—especially in the mandibular region—may have led to delayed dehiscence. Once exposed, the miniplates became susceptible to colonization by oral microorganisms and salivary biofilms. This highlights the importance of careful postoperative monitoring and soft tissue management, as even initially successful healing can be compromised over time, increasing the risk of infection and the need for implant removal.

## Discussion

This study examined osteosynthesis-associated infections (OAI) that require implant removal, potentially jeopardizing fracture stability and extending healing time, thereby elevating healthcare expenses. ^17, 18^ Conventional interventions by oral and maxillofacial surgeons often encompass drainage and fracture stabilization with antibiotics; however, hardware extraction is required in instances of osteomyelitis or non-union of bone. ^1, 14, 15^ Our data indicated a 2% incidence of OAI, consistent with the literature that cites infection rates between 2.7% to 26.8%. Infections associated with craniomaxillofacial (CMF) hardware occur less frequently than those related to extremities osteosynthesis, probably owing to the distinctive anatomy and robust blood supply of the facial region. Nonetheless, the mandible demonstrated markedly greater vulnerability to infection, as previously shown. ^11, 12, 17– 19^

This study evaluated the impact of mechanical surface damage on bacterial adhesion to titanium miniplates, revealing species-specific patterns that carry significant implications for infection control in maxillofacial surgery. Titanium, while praised for its biocompatibility and durability, is susceptible to microstructural alterations when bent, twisted, or subjected to loading—routine occurrences during intraoperative plate adaptation. These changes influence surface roughness and wettability, factors well known to facilitate bacterial attachment and biofilm formation, particularly among gram-positive organisms. ^2, 3, 15, 16, 19, 20^

Among the tested strains, *Staphylococcus aureus* exhibited the highest adherence, followed by *Pseudomonas aeruginosa*, *Enterococcus faecalis*, and *Streptococcus mutans*. These disparities likely reflect the organisms’ inherent structural and functional differences: *S. aureus* and *S. mutans* are adept at exploiting irregular, hydrophobic surfaces to initiate biofilm growth, while *P. aeruginosa*—a motile organism—may prefer smoother, hydrated substrates for initial colonization. ^15, 16, 19, 20^ This could explain its lower adhesion on damaged plates observed in our study.

Mechanical forces during surgery and mastication exacerbate these micro-imperfections. In mandibular plates, these stresses lead to surface degradation and increased hydrophilicity—both of which promote bacterial retention. ^8, 9, 12, 13, 20, 21^ This aligns with previous reports, including Zirk et al., who noted that osteosynthesis-associated infections (OAI) were more common in the mandible than in the midface, particularly with thicker or load-bearing implants. ^20, 21^ Gram-negative species such as *P. aeruginosa* and *E. faecalis* were more frequently isolated from such hardware.

Implant design is equally relevant. Mandibular plates are typically thicker (1.0 mm) than maxillary ones (0.5 mm), increasing the available surface area for bacterial colonization and the likelihood of surface damage during adaptation. Thicker plates may also exert more pressure on adjacent tissues, potentially causing ischemia and impairing immune defenses. ^5, 6, 20, 21^ In our previous study, these findings were substantiated by SEM imaging, contact angle analysis, and spectrophotometric quantification of biofilm formation. ^15, 16^

Host factors further modulate infection risk. Poor periodontal health is a recognized contributor to infection, especially in intraoral surgeries. ^8, 9^ Moreover, subclinical colonization is not uncommon; Knabl et al. showed that up to 50% of explanted plates from asymptomatic patients harbored biofilms. ^12, 13^ These findings underscore the importance of oral hygiene optimization and regular surveillance post-surgery.

Host-related factors play a critical role in implant-associated infection risk. Poor periodontal health significantly increases susceptibility, particularly in intraoral procedures, while studies show that biofilms may form even on asymptomatic hardware. ^8, 9, 12, 13^ These findings emphasize the need for optimized oral hygiene and diligent postoperative monitoring. Clinically, effective prevention should focus on gentle plate handling, judicious use of antimicrobial coatings (e.g., selenium), timely plate removal in high-risk cases, and comprehensive perioperative periodontal care. Although antibiotics remain a cornerstone, extended use beyond 24 hours offers limited benefit and may contribute to resistance, reinforcing the need for validated alternatives like selenium-coated implants. ^21– 23^

The most often found isolated bacteria were Streptococcus spp., Prevotella spp., and Staphylococcus spp., indicating their significant roles in oral wound infections. ^11, 12, 23, 24^ Pseudomonas aeruginosa was infrequently found, although it was considerably present in OAI cases associated with greater capacity plates. Infections caused by Streptococcus spp., Prevotella spp., and Staphylococcus spp. were more frequently linked to smaller volume plates, underscoring the intricate nature of osteoarticular infections, often facilitated by polymicrobial biofilms. This intricacy requires a comprehensive treatment strategy, encompassing antibiotics, debridement, wound management, and, in certain instances, hardware extraction. ^3, 4, 24– 27^

The choice of bacterial strains (S. mutans ATCC 25175, P. aeruginosa, S. aureus ATCC 25933, and E. faecalis) for inoculation in infected miniplates fulfills several research objectives. The well-characterized reference strains, sourced from esteemed culture collections (ATCC), provide consistency and repeatability in experiments. ^27, 28^ Each strain signifies a clinically relevant pathogen recognized for inducing infections in osteosynthesis, providing critical insights into infection processes and therapeutic approaches. ^27– 29^ The variety of these strains—encompassing both Gram-positive (e.g., S. mutans, S. aureus, E. faecalis) and Gram-negative (e.g., P. aeruginosa) bacteria—enables researchers to investigate different facets of biofilm development and antibiotic resistance. ^27– 30^ Moreover, employing several bacterial strains facilitates comparative analysis, enabling researchers to evaluate the effectiveness of treatments, such as antimicrobial drugs or surface coatings, against diverse pathogens, hence improving the relevance and consistency of research findings. ^23, 24, 27, 28^

Bacterial adherence to titanium miniplates differed, with S. aureus demonstrating the greatest attachment, succeeded by P. aeruginosa, E. faecalis, and S. mutans. The disparities may be ascribed to the mechanical properties and surface attributes of the miniplates. ^30, 31^ Surface imperfections, like microfractures or irregularities, might foster conditions favorable for bacterial adherence. *Staphylococcus aureus*, recognized for its adhesive characteristics and biofilm formation abilities, presumably exploits these compromised surfaces, leading to increased colonization rates relative to other bacterial strains. The thickness of titanium miniplates may affect bacterial attachment, as thicker materials offer increased surface area and imperfections that facilitate bacterial colonization. Mechanical forces during implantation and subsequent motion might induce surface deformation, hence promoting bacterial adhesion. ^31– 34^ Comprehending these pathways is crucial for formulating strategies to reduce bacterial colonization and biofilm development, hence enhancing outcomes in orthognathic surgery and facial reconstruction. ^34– 37^

The bending, twisting, and continuous loading of miniplates result in surface imperfections and degradation, facilitating bacterial adherence. The mechanical stresses, along with fluctuations in bone density in the mandible, lead to stress concentration and surface degradation, impacting the wettability and roughness of miniplates, both of which are directly associated with biofilm formation. Consequently, mandibular miniplates exposed to bending are expected to demonstrate increased hydrophilicity, offering insights into the mechanics of biofilm formation. ^31– 33, 36, 37^

Anatomical variables additionally affect the correlation between surface damage and bacterial adhesion. The mandible’s intricate biomechanics and constant mobility complicate miniplate attachment, resulting in increased vulnerability to surface injury. The acute fracture angles frequently observed in mandibular fractures exacerbate the challenges of miniplate installation and heighten the potential of surface injury. Conversely, although bending pressures exert influence on maxillary miniplates, especially in buttress regions, surface degradation is less evident than in mandibular miniplates. These structural variations must be taken into account when evaluating infection susceptibility and formulating preventive strategies in orthognathic surgery and facial reconstruction. ^37, 38^

The design of thicker miniplates in the mandible (1 mm) relative to the maxilla (0.5 mm) is noteworthy. Thicker miniplates provide an expanded surface area for bacterial colonization, which may result in enhanced biofilm formation. Moreover, bigger miniplates impose increased mechanical stress on adjacent tissues, perhaps leading to tissue damage or ischemia, so undermining the host’s immune response and facilitating biofilm development. The material composition and surface coatings of thicker miniplates affect bacterial adhesion and biofilm formation. ^12, 13^

These variables underscore the significance of implant design in reducing the incidence of implant-associated infections. In result, our investigation elucidates the complicated interplay between bacterial colonization, implant volume, and osteosynthesis-related infections, providing significant insights for the management of these difficult clinical circumstances. ^38, 39^

This research possesses multiple limitations. Initially, its in vitro design may not completely emulate the intricacies of in vivo surroundings, excluding elements such as immune response and tissue interactions. The absence of sample size and specifics of the studied miniplates may constrain statistical power. The study exclusively examined titanium miniplates, neglecting any variations in bacterial adhesion on alternative materials. It also lacks longitudinal follow-up to evaluate the evolution of bacterial populations over time. The research examined a restricted selection of bacterial strains and did not investigate differences in implant design, geometry, or coatings. Moreover, it failed to include clinical parameters, such as patient comorbidities or immunological status, that could affect infection outcomes. The influence of the host immunological response was not considered, nor were the impacts of surface imperfections and mechanical stresses comprehensively predicted. Ultimately, although bacterial attachment was noted, the growth and maturity of biofilms were not thoroughly investigated, constraining the comprehension of biofilm resistance to therapy.

## Conclusion

Our findings emphasize the intricate relationship between implant surface properties, anatomical site, and microbial behavior. Future investigations should include in vivo models with host-immune dynamics, explore biofilm-resistant biomaterials such as PEEK or selenium-coated titanium, and assess longitudinal outcomes across implant types and locations. By integrating material science with surgical technique and clinical monitoring, we can improve outcomes in orthognathic and trauma-related reconstruction.

## Ethics approval

This study was approved by the Ethics Committee of the Faculty of Dentistry – Prof. Soedomo Dental Hospital, Universitas Gadjah Mada on July 16, 2024, with a number: 150/UN1/KEP/FKG-RSGM/EC/2024. In addition, this study adheres to the Declaration of Helsinki ( https://www.wma.net/policies-post/wma-declaration-of-helsinki-ethical-principles-for-medical-research-involving-human-subjects/).

## CRediT authorship contribution statement

Conceived and designed the experiments: BPS TH. Analyzed the data: BPS AK. Wrote the paper: BPS TH. Designed search strategies: BPS AK TH. Critically reviewed the manuscript for important intellectual content: BPS AK MGW TH. Read and approved the final version: BPS AK MGW TH. Guarantors: BPS TH.

## Consent

Informed written consent was acquired from all individual participants or their guardians if they are children. Furthermore, valid informed consent was acquired from all individual participants for the publication of their data.

## Data Availability

Raw underlying data: https://doi.org/10.5281/zenodo.14728347. ^40^ Data are available under the terms of the Creative Commons Attribution 4.0 International license (CC-BY 4.0). Reporting guidelines, STROBE checklist: https://doi.org/10.5281/zenodo.14610150. ^41^ Data are available under the terms of the Creative Commons Attribution 4.0 International license (CC-BY 4.0).
