# Supplementary material for: The Influence of Surface Damage on Miniplates: A Study of Bacterial Attachment Across Various Strains
Source: F1000Res. 2025 May 21;14:158. Originally published 2025 Feb 4. [Version 2] doi: 10.12688/f1000research.159954.2 (PMC11966096; doi:10.12688/f1000research.159954.2)
Supplement: Peer review history

# Reviewer report: Reviewer response for version 1

**DOI:** 10.5256/f1000research.175749.r371018

**Published:** 2025-04-02

**Authors:** Anupam Singh

**Affiliations:** 1 Manipal College of Dental Sciences, Manipal, India

1. Abstract - Authors emphasise the application of miniplates in orthognathic surgery, however, the manuscript pertains mainly to the trauma patients and even the inclusion criteria cites the patients with simple fractures.

 2. Kindly mention about the type of study - prospective/retrospective, observational/longitudinal in the methdology

 3. How were the mini plates retrieved? How were they transferred from the operating room to the lab? Were they stored in any storage medium?

 4. What were the parameters of 'control plates'? Were these the plates that were never exposed to any external environment?

 5. 'The design of thicker miniplates in the mandible (1 mm) relative to the maxilla (0.5 mm) is noteworthy.' Elsewhere the authors have mentioned about the use of 1.6mm and 2.0mm miniplates. While here authors mention 1.0mm plates for mandible and 0.5mm plates for maxilla.

 6. Was there any local wound dehiscence noted? What is the rationale for exposure of miniplates to oral secretions if the wound healing is adequate in the initial healing phase? It would be beneficial for the readers if the authors can discuss about this particular aspect.

 7. The latest articles cited have been from 2020. Authors are advised to cite some recent relevant articles as well.

 8. A section of discussion can be written about the ways of mitigating the infection apart from eliminating or reducing the miniplate bending

Is the work clearly and accurately presented and does it cite the current literature?

Partly

If applicable, is the statistical analysis and its interpretation appropriate?

I cannot comment. A qualified statistician is required.

Are all the source data underlying the results available to ensure full reproducibility?

Partly

Is the study design appropriate and is the work technically sound?

Yes

Are the conclusions drawn adequately supported by the results?

Yes

Are sufficient details of methods and analysis provided to allow replication by others?

Partly

Reviewer Expertise:

Oral and Maxillofacial surgery

I confirm that I have read this submission and believe that I have an appropriate level of expertise to confirm that it is of an acceptable scientific standard, however I have significant reservations, as outlined above.

## References

[1] : Reasons for Removal of Miniplates Used in Fixation of Maxillofacial Bone Fractures: Systematic Review and Meta-Analysis.2023; 10.20944/preprints202309.1497.v1 10.20944/preprints202309.1497.v1

[2] : Reasons for Removal of Miniplates Used in Fixation of Maxillofacial Bone Fractures: Systematic Review and Meta-Analysis. *Applied Sciences* .2023;13(21) : 10.3390/app132111899 10.3390/app132111899

[3] : Evaluation of Selenium Coating on Titanium Miniplates and Screws – an In Vitro Study.2024; 10.20944/preprints202411.0824.v1 10.20944/preprints202411.0824.v1

# Reviewer report: Reviewer response for version 1

**DOI:** 10.5256/f1000research.175749.r371021

**Published:** 2025-03-28

**Authors:** Mohamed Jaber

**Affiliations:** 1 Ajman University, Ajman, Ajman, United Arab Emirates

**Reviewer Report**

**Title:** The Influence of Surface Damage on Miniplates: A Study of Bacterial Attachment Across Various Strains.

**1. Title**

 The title is clear, concise, and accurately reflects the study's focus. It highlights key variables (surface damage, bacterial attachment) and the scope (various bacterial strains). However, specifying the type of miniplates (e.g., titanium) could enhance precision.

**2. Abstract**

 The abstract follows a standard structure (Background, Methods, Results, Conclusion) and succinctly summarizes the study. However:

- Clearly states the problem, methodology, and key findings.
- Lacks specific quantitative results (e.g., adhesion rates, p-values). The conclusion could briefly mention clinical implications.

**3. Introduction **

- Clearly outlines the clinical problem (postoperative infections due to miniplate damage).
- Cites relevant studies on bacterial biofilms and osteosynthesis infections.
- Some citations are outdated (e.g., 2008, 2011); more recent literature (last 5 years) should be included.
- The gap in knowledge (impact of surface damage on bacterial adhesion) is stated but could be emphasized more explicitly.
- The study aims are clearly defined: to evaluate bacterial attachment on damaged miniplates. However, the objectives could be more specific (e.g., quantifying adhesion differences between strains).

- Several citations are >10 years old. Recent studies (e.g., on biofilm-resistant materials) should be included.
- The study identifies gaps (e.g., impact of mechanical stress on bacterial adhesion) but could propose specific future research directions (e.g., in vivo models).

- The review covers key concepts but lacks depth in recent advances in antimicrobial coatings for miniplates and comparative studies on different miniplate materials (e.g., PEEK vs. titanium).
- The aims and objectives were explicitly stated, concise, and achievable. They align with the research question and methodology.

**4. Methodology**

- Robust techniques (SEM, spectrophotometry, contact angle measurements).
- Standardized bacterial cultures (McFarland turbidity).
- No details on how surface damage was quantified (e.g., roughness metrics).
- Limited discussion of potential confounding factors (e.g., patient-specific variables).

**5. Result**

- Figures are well-labeled but lack error bars in graphs (e.g., Figure 4).
- SEM images are high-quality but could benefit from scale bars and annotations.

- Non-parametric tests (Kruskal-Wallis, Mann-Whitney) are appropriate for non-normal data. However, no justification for not using parametric tests (e.g., Shapiro-Wilk test for normality).
- Post-hoc corrections for multiple comparisons are not mentioned.

**6. Discussion**

- The discussion should compare the study’s findings with existing literature. This helps in contextualizing the results and highlighting their significance.
- Limited comparison to similar studies (e.g., conflicting results in literature).
- Incomplete discussion of limitations (e.g., in vitro vs. in vivo conditions).
- Links findings to clinical implications (e.g., mandibular miniplates higher risk).
- Discusses bacterial strain-specific adhesion patterns.
- Does not fully explain why *P. aeruginosa* adhered less to damaged plates.
- The conclusion aligns with results but overgeneralizes findings (e.g., "further research is needed" without specifics). Clinical recommendations (e.g., miniplate handling protocols) could be added.

**7. Overall assessment**

     Overall, the study is well-designed, but some areas need improvement:

- Comprehensive methodology (SEM, contact angle measurements, bacterial assays).
- Clear inclusion/exclusion criteria for miniplate samples.
- Small sample size (12 miniplates from 10 patients), limiting generalizability.
- No discussion of effect sizes or confidence intervals.
- Lack of negative controls in bacterial attachment assays.
- Lack of quantitative surface damage analysis.
- Language is clear but occasionally verbose (e.g., repetitive descriptions of SEM methods). The discussion could better contrast results with prior studies (e.g., why *S. aureus* adhered more than *P. aeruginosa*).

**8. Plagiarism Check**

 No overt plagiarism detected, but some passages are overly similar to cited literature (e.g., descriptions of biofilm formation). Paraphrasing could improve originality.

**9. Suitability for Publication**

 The manuscript present study of moderate significance for clinical practice, but methodological clarity and literature updates are needed).

 However, the manuscript is suitable after **major revisions**, more specifically:

- Clarify statistical methods and add effect sizes.
- Include recent literature (last 5 years).
- Strengthen the discussion with comparative analysis.
- Shorten redundant methodology descriptions.
- Add quantitative metrics for surface damage (e.g., roughness measurements).
- Discuss limitations of in vitro vs. in vivo biofilm formation.
- Include recent studies on antimicrobial miniplate coatings.
- Simplify methods section to avoid redundancy.
- Propose specific future research directions in the conclusion.

Is the work clearly and accurately presented and does it cite the current literature?

Partly

If applicable, is the statistical analysis and its interpretation appropriate?

Partly

Are all the source data underlying the results available to ensure full reproducibility?

Yes

Is the study design appropriate and is the work technically sound?

Yes

Are the conclusions drawn adequately supported by the results?

Yes

Are sufficient details of methods and analysis provided to allow replication by others?

Partly

Reviewer Expertise:

Maxillofacial trauma, Oral cancer and Precancer, Dental education, Salivary gland

I confirm that I have read this submission and believe that I have an appropriate level of expertise to confirm that it is of an acceptable scientific standard, however I have significant reservations, as outlined above.

# Reviewer report: Reviewer response for version 1

**DOI:** 10.5256/f1000research.175749.r371017

**Published:** 2025-03-22

**Authors:** faizul hasan

**Affiliations:** 1 Faculty of Nursing Chulalongkorn University, Thailand, Thailand

Your work provides a coherent analysis with clinical significance, delivering important insights into the subject field.

 The study clearly elucidates significant findings and their significance for clinical practice, but certain portions may benefit from more succinct explanations.

 Enhancing the discourse by establishing a more explicit link to existing literature and actual implementations would amplify its influence.

 Moreover, maintaining uniformity in terminology and enhancing methodological descriptions could augment clarity. The research significantly contributes to the area; yet, simple enhancements might improve its clarity and therapeutic relevance.

Is the work clearly and accurately presented and does it cite the current literature?

Yes

If applicable, is the statistical analysis and its interpretation appropriate?

Yes

Are all the source data underlying the results available to ensure full reproducibility?

Yes

Is the study design appropriate and is the work technically sound?

Yes

Are the conclusions drawn adequately supported by the results?

Yes

Are sufficient details of methods and analysis provided to allow replication by others?

Yes

Reviewer Expertise:

Dental Medicine, Oral Biology, Dental Pharmacology

I confirm that I have read this submission and believe that I have an appropriate level of expertise to confirm that it is of an acceptable scientific standard.
